# A review of the taxonomy of spiny-backed orb-weaving spiders of the subfamily Gasteracanthinae (Araneae, Araneidae) in Thailand

**DOI:** 10.3897/zookeys.1032.62001

**Published:** 2021-04-16

**Authors:** Kongkit Macharoenboon, Warut Siriwut, Ekgachai Jeratthitikul

**Affiliations:** 1 Animal Systematics and Molecular Ecology Laboratory, Department of Biology, Faculty of Science, Mahidol University, Bangkok 10400, Thailand Animal Systematics and Molecular Ecology Laboratory, Department of Biology, Faculty of Science, Mahidol University Bangkok Thailand

**Keywords:** Gasteracanthinae, molecular phylogeny, species delimitation, taxonomy, Thailand

## Abstract

Spiny-backed orb-weaving spiders of the subfamily Gasteracanthinae are broadly distributed in the Old World. Despite their use as a model species in biology, evolution, and behavior because of their extraordinary characteristics, the systematics of this group of spiders are still poorly understood. This study elucidates the systematics of Gasteracanthinae in Thailand based on morphological and molecular-based analyses. In total, seven species from three genera, namely *Gasteracantha*, *Macracantha*, and *Thelacantha*, were recorded in Thailand. Shape of abdominal spines, pattern of sigilla, and female genitalia are significant characters for species identification. In contrast, coloration shows highly intraspecific variation in most species within Gasteracanthinae. A phylogenetic tree based on partial sequences of COI, 16S, and H3 genes recovered Gasteracanthinae as a monophyletic group and supports the existence of three clades. *Gasteracantha hasselti* is placed as a sister taxon to *Macracantha arcuata*. Hence, we propose to transfer *G. hasselti* to *Macracantha.* Moreover, molecular species delimitation analyses (ABGD, bPTP, and GMYC) using 675 bp of COI gene support all nominal species, with evidence of possible additional cryptic species.

## Introduction

Spiny-backed orb-weaving spiders are a group of spiders typically featuring an abdomen decorated with conspicuous spines and of notable coloration (Pickard-Cambridge 1879; Simon 1892; Dahl 1914; Emerit 1974; Barrion and Litsinger 1995). These spiders are currently classified into two subfamilies, Micratheninae and Gasteracanthinae (Scharff and Coddington 1997). Micratheninae is widespread in the New World, while most species within Gasteracanthinae are broadly distributed in the Old World (Scharff and Coddington 1997). It has been suggested that the abdominal spines of members of both Gasteracanthinae and Micratheninae serve as defensive structures (Peckham 1889; Cloudsley-Thompson 1995), whereas the distinct coloration possibly plays a role in prey attraction (Kemp et al. 2013) and aposematism (Cloudsley-Thompson 1995). Some species also exhibit intraspecific color polymorphism, for example, *Gasteracantha cancriformis* (Linnaeus, 1758), *Gasteracantha fornicata* (Fabricius, 1775), and *Thelacantha brevispina* (Doleschall, 1857) (Truong 2012; Kemp et al. 2013; Salgado-Roa et al. 2018; Chamberland et al. 2020). This polymorphism suggests different adaptive advantages for each morph and/or the effect of frequency-dependent selection (Punzalan et al. 2005; Ishii and Shimada 2010; Cotoras et al. 2016; White and Kemp 2016). Moreover, these spiders are well known for their sexual dimorphism: the males are extremely reduced in size, and their spines are poorly developed (Pickard-Cambridge 1879; Hormiga et al. 2000). Due to such extraordinary characteristics of Gasteracanthinae, they are frequently used as species models for evolutionary, biological, ecological, and behavioral studies (i.e., Yoshida 1989; Jaffé et al. 2006; Gawryszewski and Motta 2012; Kemp et al. 2013).

The taxonomy of Gasteracanthinae was first proposed by Simon in 1892. The author placed almost all old-world spiny-backed orb-weavers in the tribe Gasteracantheae, which feature distinct morphological characters, i.e., a hard-sclerotized abdomen that overlaps the cephalothorax, the presence of conspicuous sigilla on dorsal abdomen, and prominent abdominal spines (Simon 1892). Subsequently, Dahl (1914) classified *Gasteracantha*, the predominant genus in Gasteracanthinae, into 16 subgenera based on the shape and position of abdominal spines, structure of abdomen, and sigilla pattern. Since then, several subgeneric names or junior synonyms of *Gasteracantha* have been revalidated (Benoit 1962, 1964; Emerit 1974). Scharff and Coddington (1997) reconstructed the phylogeny of Araneidae and revealed that spiny-backed orb-weaving spiders did not represent a monophyletic group, but were instead separated into two monophyletic clades, consisting of species from the Old World and New World, respectively. Based on these results, the authors classified all new-world genera into subfamily Micratheninae, and placed *Gasteracantha* and the rest of the old-world genera in subfamily Gasteracanthinae. The distant relationship between Micratheninae and Gasteracanthinae was later supported by several molecular and transcriptomic studies (Álvarez-Padilla et al. 2009; Dimitrov et al. 2017; Wheeler et al. 2017; Fernández et al. 2018; Kallal et al. 2018; Scharff et al. 2020).

Thailand is located within two significant biodiversity hotspots, Indo-Burma and Sundaland, and is home to a high biodiversity of flora and fauna (Myers et al. 2000). At the time of writing, 43 species of spiders from four genera of Gasteracanthinae (*Actinacantha* Simon, 1864; *Gasteracantha* Sundevall, 1833; *Macracantha* Fabricius, 1793; and *Thelacantha* Hasselt, 1882) have been recorded in Southeast Asia, of which ten species from three genera were recorded in Thailand (World Spider Catalog 2020), including *Gasteracantha clavigera* Giebel, 1863; *Gasteracantha diadesmia* Thorell, 1887; *Gasteracantha diardi* (Lucas 1835); *Gasteracantha frontata* Blackwall, 1864; *Gasteracantha irradiata* (Walckenaer, 1841); *Gasteracantha kuhli* C. L. Koch, 1837; *Gasteracantha hasselti* C. L. Koch, 1837; *Gasteracantha rubrospinis* Guérin, 1838; *Macracantha arcuata* (Fabricius, 1793); and *Thelacantha brevispina* (Doleschall, 1857). However, the taxonomy of Gasteracanthinae at the species level remains unclear because of the general scarcity of male specimens for morphological and molecular study, the lack of morphological characters for the identification of sub-adults and male spiders, and intraspecific morphological variation and morphological resemblance among closely related species (Pickard-Cambridge 1879; Dahl 1914; Tan et al. 2019). Molecular approaches in terms of DNA barcoding and species delimitation can resolve these taxonomic issues. These techniques were successfully applied in several studies of different spider groups, and can be especially helpful in differentiating among morphologically similar taxa (Zhang and Li 2014; Hedin 2015; Ortiz and Francke 2016). However, molecular data of Gasteracanthinae in South East Asia are still lacking. Only the study by Tan et. al. (2019) has focused on phylogeny of Gasteracanthinae at species/population levels.

The objective of this study is to elucidate the taxonomy of spiny-backed orb-weavers in subfamily Gasteracanthinae, specifically in *Gasteracantha*, *Macracantha*, and *Thelacantha*, based on the morphological and molecular analyses of specimens from Thailand.

## Materials and methods

### Specimen sampling

Spiders were collected throughout Thailand by visual searching in several types of habitats, including rainforest, dipterocarp forest, paddy field, mangrove forest, and areas with human development. Specimens were euthanized following methods of Cooper (2011). Animal use in this study was approved by the Faculty of Science, Mahidol University Animal Care and Use Committee SCMU-ACUC (MUSC62-002-466). All specimens were preserved in 95% (v/v) ethanol and kept at -20 °C for molecular work and long-term storage. The dorsal and ventral views of each morphotype were photographed using Nikon D7200 + Nikon AF-S Nikkor 105 mm f/2.8G IF-ED VR Micro. All voucher specimens were deposited at Mahidol University of Natural History Museum, Salaya, Thailand (**MUMNH**).

### Species identification

Species identification was primarily based on external and internal morphology, with emphasis on the characteristics of shape and position of abdominal spines, color pattern on abdomen, sigilla pattern, and epigynal structure. The morphology of each species was examined by using complete adult female specimens. Previous taxonomic publications including original descriptions were used as reference for species identification (Simon 1877; Pickard-Cambridge 1879; Thorell 1887; Pocock 1900; Dahl 1914; Emerit 1974; Tikader 1982; Barrion and Litsinger 1995; Sen et al. 2015; Roy et al. 2017). In order to observe female reproductive organs, the ventral plate was removed using an insect pin. It was immersed in saturated 5% (v/v) KOH for 5–10 minutes to clean off remaining soft tissue, then washed in distilled water. Internal and external morphology of specimens was observed under Nikon stereoscopic zoom microscope SMZ745. All measurements were taken from the left side of the body and recorded in millimeters. Leg measurements are provided as total length (femur, patella, tibia, metatarsus, and tarsus).

Abbreviations for female genitalia are: **S** = spermatheca, **CD** = copulatory duct, **FD** = fertilization duct, **EF** = epigastric furrow, **UP** = upper patch (the sclerotized plate on the top of epigynum), and **SC** = scape.

### Molecular analyses

A total of 32 individuals were selected. Fragments of two mitochondrial genes, Cytochrome c oxidase subunit I (COI) and 16S rRNA (16S); and one nuclear marker, Histone subunit 3 (H3) were amplified as molecular markers. Genomic DNA was extracted from four right legs of each spider by using NucleoSpin tissue kit (MACHEREY-NAGEL, Germany). Primer sets used for PCR amplification are summarized in Table 1. PCR reactions were performed using T100 thermal cycle (Bio-Rad Laboratories, USA) with the following conditions: 5 min at 94 °C; 30 cycles of denaturation for 60 s at 94 °C, annealing for 45 s at 48–51 °C, and elongation for 90 s at 72 °C; pre-denaturation for 3 min at 94 °C, and post-elongation for 4 min at 72 °C. The final total PCR volume was 30 µl, consisting of 15 µl of EmeraldAmp PCR Maser Mix (TAKARA BIO INC.), 1.5 µl of both forward and reverse primers, 9 µl of distilled water, and 3 µl of template DNA (at least 25 ng). PCR products were checked by running a 1.5% agarose gel electrophoresis, and were purified by PEG precipitation. Purified samples were sequenced by Sanger method using automated sequencer (ABI prism 3730XL).

**Table 1.** Primers used for the PCR reaction and sequencing in this study.

Genes	Primer	Reference
COI	LCO-1490: 5'-GGT CAA CAA ATC ATA AAG ATA TAT TGG-3'	Folmer et al. (1994), Simon et al. (1994)
	NANCY: 5'-CCC-GGT-AAA-ATT-AAA-ATA-TAA-ACT-TC-3'	
16S	16Sa: 5'-CGC-CTG-TTT-ATC-AAA-AAC-AT-3'	Palumbi et al. (1991)
	16Sb: 5'-CTC-CGG-TTT-GAA-CTC-AGA-TCA-3'	
H3	H3aF: 5'-ATG-GCT-CGT-ACC-AAG-CAG-ACV-GC-3'	Colgan et al. (1998)
	H3aR: 5'-ATA-TCC-TTR-GGC-ATR-ATR-GTG-AC-3'	

### Phylogenetic analyses

Sequences were automatically aligned in MEGA X (Kumar et al. 2018) using MUSCLE alignment (Edgar 2004), then manually checked and edited. Edited sequences were deposited in GenBank; the accession numbers and related information are summarized in Table 2.

**Table 2.** Samples used in this study, with specimen vouchers and GenBank accession numbers.

**species**	**Voucher**	**Locality**	**Accession number**			**Reference**
			**COI**	**16S**	**H3**	
*Gasteracantha diadesmia*	MUMNH-ARA-GAS011	Nakhon Ratchasima, Thailand	MT584892	MT584924	-	This study
	MUMNH-ARA-GAS047	Mae Hong Sorn, Thailand	MT584893	MT584925	MT584953	This study
	MUMNH-ARA-GAS067	Surat Thani, Thailand	MT584894	MT584926	MT584954	This study
	MUMNH-ARA-GAS117	Loei, Thailand	MT584895	MT584927	MT584955	This study
*Gasteracantha diardi*	MUMNH-ARA-GAS021	Chumpon, Thailand	MT584896	MT584928	MT371076	This study
	MUMNH-ARA-GAS104	Nakhon Si Thammarat, Thailand	MT584897	MT584929	MT584956	This study
	MUMNH-ARA-GAS127	Phumi Pôpôk Vil, Cambodia	MT584898	MT584930	MT584957	This study
	MUMNH-ARA-GAS129	Chiangmai, Thailand	MT584899	MT584931	-	This study
	MUMNH-ARA-GAS132	Nakhon Ratchasima, Thailand	MT584900	MT584932	-	This study
	GDIA1	Kedah, Malaysia -	KU055841	KU055746	MG670171	Tan et al. 2019
	GDIA3	Penang, Malaysia	MG670114	MG670142	MG670173	Tan et al. 2019
*Gasteracantha doriae*	MUMNH-ARA-GAS053	Trat, Thailand	MT584901	MT584933	MT584958	This study
	MUMNH-ARA-GAS068	Suratthani, Thailand	MT584902	MT584934	MT584959	This study
	MUMNH-ARA-GAS130	Rayong, Thailand	MT584890	MT584922	MT584951	This study
	MUMNH-ARA-GAS131	Rayong, Thailand	MT584891	MT584923	MT584952	This study
	GDIA5	Perak, Malaysia	MG670116	MG670144	MG670175	Tan et al. 2019
	GDIA6	Perak, Malaysia	MG670117	MG670145	MG670176	Tan et al. 2019
*Gasteracantha kuhli*	MUMNH-ARA-GAS007	Surat Thani, Thailand	MT584909	MT584941	-	This study
	MUMNH-ARA-GAS029	Ratchaburi, Thailand	MT584910	MT584942	MT371077	This study
	MUMNH-ARA-GAS033	Samut Prakan, Thailand	MT584911	MT584943	MT584962	This study
	MUMNH-ARA-GAS042	Krabi, Thailand	MT584912	MT584944	MT584963	This study
	MUMNH-ARA-GAS101	Chiangmai, Thailand	MT584913	MT584945	-	This study
	GKUH2	Selangor, Malaysia	MG670118	MG670146	MG670177	Tan et al. 2019
	GKUH3	Pahang, Malaysia	MG670119	MG670147	MG670178	Tan et al. 2019
*Gasteracantha cancriformis*	787198	Hispaniola	KJ157212	KJ156989	-	McHugh et al. 2014
	782149	Puerto Rico	KJ157214	KJ156990	-	McHugh et al. 2014
	N/A	N/A	FJ525321	FJ525354	FJ525340	Agnarsson and Blackledge 2009
*Macracantha arcuata*	MUMNH-ARA-MAC005	Krabi, Thailand	MT584914	MT584946	MT584964	This study
	MUMNH-ARA-MAC008	Prachuab Khiri Khan, Thailand	MT584915	MT584947	MT584965	This study
	Mar-02	Selangor, Malaysia	MG670122	MG670150	MG670181	Tan et al. 2019
	Mar-03	Kedah, Malaysia	MG670123	MG670151	MG670182	Tan et al. 2019
	ZMUC00008513	Naknon Sri Thammarat, Thailand	MK420123	MK420239	MK420339	Scharff et al. 2020
	MUMNH-ARA-MAC011	Chiangmai, Thailand	MT584916	MT584948	MT584966	This study
	MUMNH-ARA-MAC021	Phumi Pôpôk Vil, Cambodia	MT584917	MT584949	MT584967	This study
*Macracantha hasselti*	MUMNH-ARA-GAS016	Ubon Ratchathani, Thailand	MT584903	MT584935	-	This study
	MUMNH-ARA-GAS018	Saraburi, Thailand	MT584904	MT584936	MT371075	This study
	MUMNH-ARA-GAS025	Phetchaburi, Thailand	MT584905	MT584937	-	This study
	MUMNH-ARA-GAS037	Phetchaburi, Thailand	MT584906	MT584938	MT584960	This study
	MUMNH-ARA-GAS050	Mae Hong Sorn, Thailand	MT584907	MT584939	MT584961	This study
	MUMNH-ARA-GAS065	Chumpon, Thailand	MT584908	MT584940	-	This study
	GHAS1	Kedah, Malaysia	MG670120	MG670148	MG670179	Tan et al. 2019
*Thelacantha brevispina*	MUMNH-ARA-THE004	Phetchaburi, Thailand	MT584918	-	MT584968	This study
	MUMNH-ARA-THE005	Surat Thani, Thailand	MT584919	-	MT584969	This study
	MUMNH-ARA-THE007	Loei, Thailand	MT584920	-	MT584970	This study
	MUMNH-ARA-THE008	Samut Prakan, Thailand	MT584921	MT584950	MT584971	This study
	TBRE1	Penang, Malaysia	MG670124	MG670152	MG670183	Tan et al. 2019
	TBRE2	Penang, Malaysia	MG670125	MG670153	MG670184	Tan et al. 2019
	TBRE3	Kedah, Malaysia	MG670126	MG670154	-	Tan et al. 2019
	sc06156	French Polynesia	KX055041	-	-	Ramage et al. 2017
	sc05514	French Polynesia	KX055044	-	-	Ramage et al. 2017
	Gam_Ok01	Okinawa, Japan	AB969824	-	-	Yamada et al. 2015
*Actinacantha globulata*	AGLO1	Semenyih, Selangor, Malaysia	MG670112	MG670140	MG670170	Tan et al. 2019
**Outgroup**						
*Cyclosa caroli*	n92	USA: Florida, Gainesville	MK420091	MK420211	MK420316	Scharff et al. 2020
*Cyclosa turbinata*	CA	USA: California, Encinitas	MK420092	MK420212	MK420317	Scharff et al. 2020
*Cyclosa walckenaeri*	n94	USA: California, Big Sur	MK420093	MK420213	MK420318	Scharff et al. 2020
*Micrathena gracilis*	102	USA: Ohio	MK420136	MK420251	MK420349	Scharff et al. 2020
*Micrathena gracilis*	N/A	N/A	FJ525326	FJ525359	FJ525343	Agnarsson and Blackledge 2009
*Micrathena horrida*	784351	Cuba	KJ157243	KJ157016	-	McHugh et al. 2014
*Micrathena sagittata*	7	USA: Florida, Gainesville, 7.vii.1997	MK420137	MK420253	-	Scharff et al. 2020
*Herennia etruscilla*	N/A	N/A	KC849074	KC849118	KC849033	Kuntner et al. 2013
*Herennia multipuncta*	N/A	N/A	KC849075	KC849119	KC849034	Kuntner et al. 2013
*Nephila pilipes*	N/A	N/A	KC849088	KC849130	KC849045	Kuntner et al. 2013
*Nephila clavate*	N/A	N/A	KC849082	KC849125	KC849041	Kuntner et al. 2013
*Nephila clavipes*	N/A	N/A	FJ525328	FJ525361	FJ525344	Agnarsson and Blackledge 2009
*Nephila senegalensis*	N/A	N/A	KC849090	KC849132	KC849047	Kuntner et al. 2013
*Nephilengys dodo*	N/A	N/A	KC849097	KC849138	KC849053	Kuntner et al. 2013
*Nephilengys malabarensis*	N/A	N/A	KC849099	KC849140	KC849055	Kuntner et al. 2013

In this study, we included sequences from previous publications as outgroups and some sequences of Gasteracanthinae from Thailand and adjacent countries as ingroups (Table 2). The outgroups were the subfamily Nephilinae, which is considered as a sister clade of the rest of Araneidae (Hormiga and Griswold 2014); genus *Micrathena*, another spiny orb-weaver from Neotropical regions; and genus *Cyclosa*, which is considered to be closely related to Gasteracanthinae (Wheeler et al. 2017). Phylogenetic analyses were conducted based on maximum parsimony (MP), maximum likelihood (ML), and Bayesian inference (BI). Each gene was independently partitioned to find the best-fit models for nucleotide substitution using KAKUSAN4 (Tanabe 2007) with Bayesian Information Criterion (BIC) (Schwarz 1978). Only COI was further partitioned by codon position into three partitions. The best-fit models for each partition were GTR+G for the first and the third codon positions of COI and for 16S; F81+G for the second codon position of COI; and HKY85+G for H3.

For MP analyses, multiple sequences were used to generate molecular matrices using GB2TNT (Goloboff and Catalano 2012). Maximum parsimonious tree was constructed with TNT v. 1.5 (Goloboff and Catalano 2016). TNT searches were run with 5,000 replications of traditional heuristic search. Trees were saved twice per replicate. Branch-swapping was conducted with tree bisection-reconnection (TBR). Support for nodes was accessed using Jackknifing (Farris 1997) with 1,000 pseudo-replications, and set character removal probability equal to 36% under the traditional search. ML analyses were executed in RAxML v.8.2.12 (Stamatakis 2014). Due to limitations in best-fit model selection in RAxML, all analyses were performed under GTRGAMMA model. Support clades were assessed with 1,000 bootstrap replications. BI analyses were performed in MRBAYES v3.2.6 (Ronquist et al. 2012) on the online CIPRES Science Gateway server (Miller et al. 2010), using Markov chain Monte Carlo (MCMC), and sampling for 20,000,000 generations. Each run contained four chains with the temperature setting of 0.05. Trees were sampled every 200 generations. The first 25% of trees were discarded as burn-in. The results of MCMC sampling were monitored using Tracer v. 1.7 (Rambaut et al. 2018) to ensure that Markov chains had run to become stationary, the standard deviation of split frequencies was below 0.01, and effective sampling size (ESS) exceeded 200 for all parameters after burn-in.

Genetic distances between species within Gasteracanthinae were examined using COI sequence (675 bp) via uncorrected pairwise genetic distance as implemented in MEGA X (Kumar et al. 2018). The examined taxa were grouped following the clusters from species delimitation results.

### Species delimitations

Species delimitations were analyzed via computational methods to examine whether each lineage (or putative species) in the phylogenetic tree was statistically significant as a distinct species. The sequence matrices of the COI gene (675 bp), 16S gene (454 bp), and H3 gene (328 bp) were used as DNA barcoding. Each dataset consisted of 52, 46, and 38 individuals, respectively. Delimitation of each taxa was executed using Automatic barcode gap discovery (ABGD), Bayesian Poisson tree processes (bPTP), and Generalized mixed Yule coalescent (GMYC). Firstly, Automatic barcode gap discovery (ABGD) analysis (Puillandre et al. 2011) was run on the online sever: https://bioinfo.mnhn.fr/abi/public/abgd/abgdweb.html. Default parameters were used, except the relative gap width, which was set at 1.0. Kimura-2-parameter was used as substitution model (Kimura 1980). Secondly, the Bayesian Poisson tree processes (bPTP) was carried out using the bPTP server: https://species.h-its.org/. The ML tree reconstructed from RAxML was used as input data (Zhang et al. 2013). The analysis was run as rooted with outgroups removed, sampling MCMC 500,000 generations, 500 of thinning, and burn-in as 0.1. Thirdly, the Generalized mixed Yule coalescent (GMYC; Pons et al. 2006) was performed using the BI tree from BEAST v.2.6.2 (Bouckaert et al. 2014) under Yule speciation model. The analysis was run for 5,000,000 generations. Trees were sampled every 1,000 generations. Sampled trees from BEAST were summarized onto a single tree using TreeAnnotator v.2.6 (BEAST package), with 25% of samples discarded as burn-in. The GMYC analysis was conducted with the ‘splits’ package using R v.3.6 (available at http://r-forge.rproject.org/projects/splits). The species delimitations by these three methods were compared for consistency with (1) morphological characters between OTUs based on original descriptions and previous taxonomic reviews, (2) uncorrected genetic distance between OTUs by using COI sequence (675 bp), and (3) molecular phylogenetic analyses based on partial sequences of COI, 16S, and H3 genes.

## Results

### Morphological study

**Figure 1.**
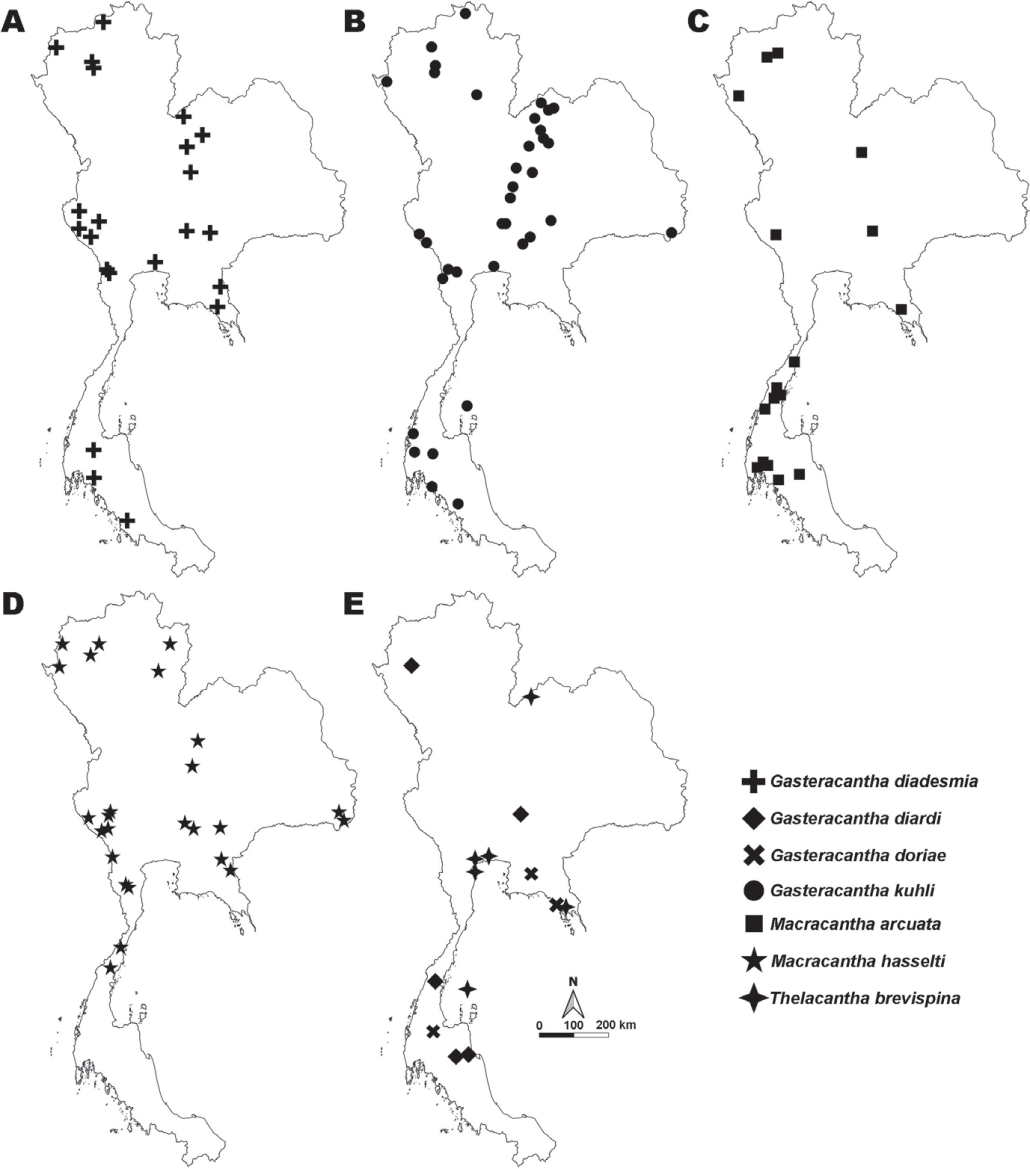
Distribution map of Gasteracanthinae in Thailand **A***Gasteracantha diadesmia***B***G. kuhli***C***Macracantha arcuata***D***M. hasselti***E***G. diardi*, *G. doriae*, and *Thelacantha brevispina*.

A total of 342 spiders from 93 localities was morphologically identified to seven species from three genera: *Gasteracantha diardi* (Lucas, 1835), *Gasteracantha diadesmia* Thorell, 1887, *Gasteracantha doriae* Simon, 1877, *Gasteracantha kuhli* Koch, 1837, *Gasteracantha hasselti* Koch, 1837, *Macracantha arcuata* (Fabricius, 1793), and *Thelacantha brevispina* (Doleschall, 1857). Distribution maps of all species are presented in Fig. 1. We were unable to obtain specimens of four species previously recorded and/or described from Thailand for this study: *Gasteracantha frontata* Blackwall, 1864, *Gasteracantha irradiata* Walckenaer, 1842, *Gasteracantha rubrospinis* Guérin, 1838, and *Gasteracantha clavigera* Giebel, 1863 (Giebel 1863; Pocock 1897; Simon 1886; Dahl 1914).

**Figure 2.**
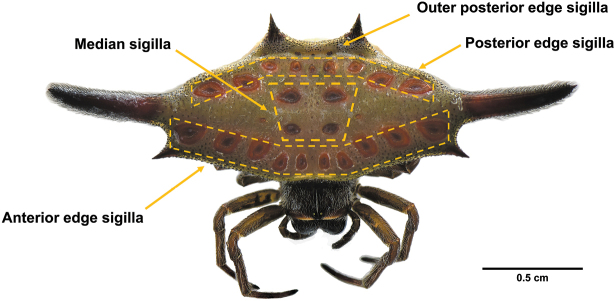
Female *Gasteracantha diardi* with proposed names of the abdominal sigilla groups used in this study.

The number of dorsal sigilla in most species is equal, but the arrangement, shape and size are variable among species. To describe the number and position of sigilla on the abdomen, we divide the abdominal sigilla into four groups according to their position (Fig. 2): (i) the anterior edge sigilla form a row near the anterior border of the dorsal abdomen, (ii) the posterior edge sigilla form a row near the posterior border of dorsal abdomen, (iii) the median sigilla are situated in the middle of the dorsal abdomen, arranged in a trapezoid shape, and (iv) the outer posterior edge sigilla form a row behind the posterior border of the dorsal abdomen.

### Phylogenetic analyses and genetic divergence

**Figure 3.**
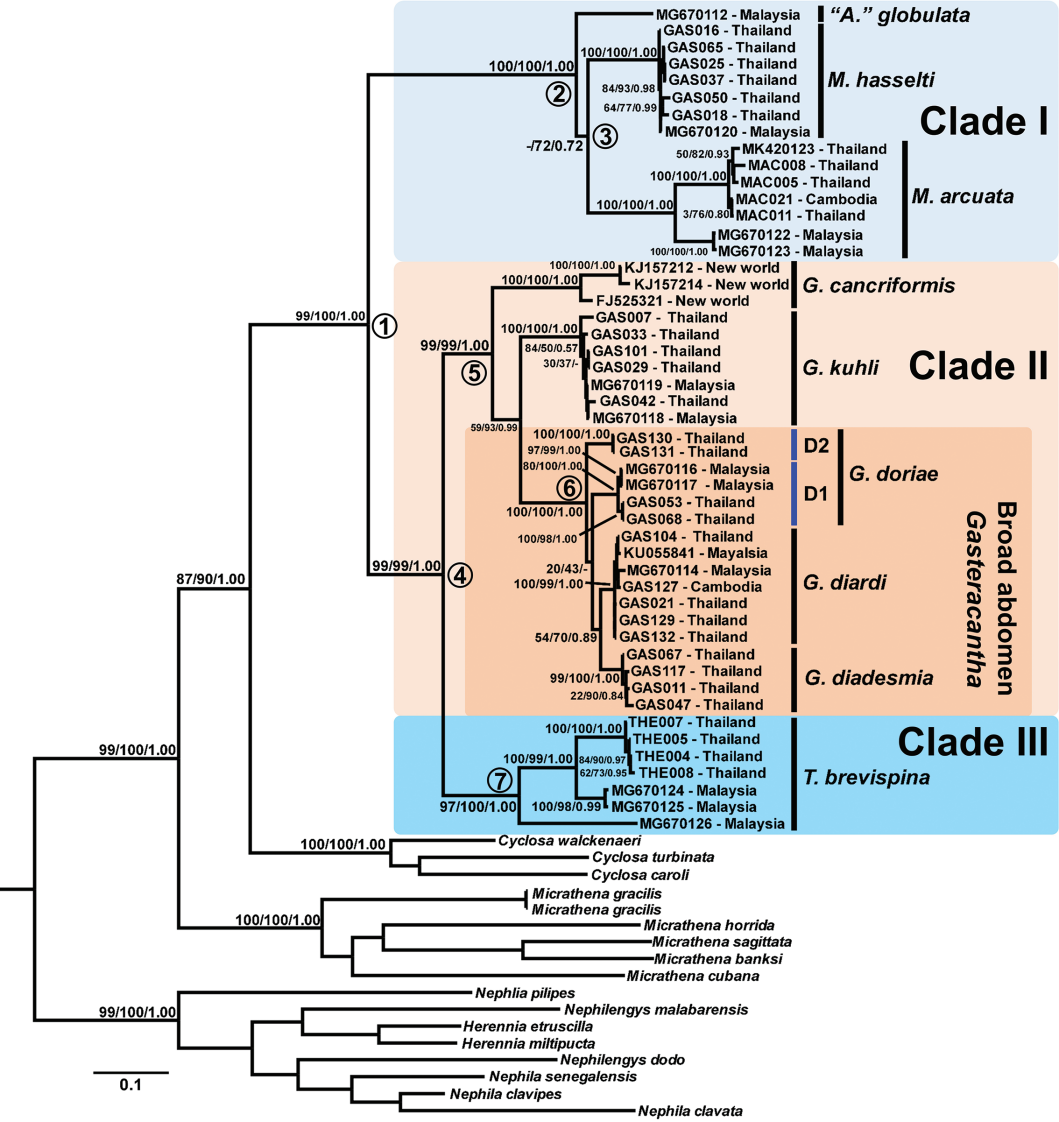
Maximum Likelihood phylogenetic tree reconstructed from COI+16S+H3 genes of Gasteracanthinae and outgroups. Nodal support values are labeled as Jackknife support/ML bootstrap values/Bayesian posterior probability.

The total length of the concatenated alignment was 1457 bp, consisting of 675 bp of COI, 454 bp of 16S rRNA and 328 bp of H3. The concatenated dataset had 288, 252, and 105 variable sites and 252, 202, and 83 parsimonious informative sites, for COI, 16S, and H3, respectively. The three phylogenetic methods recovered some differences in branching patterns. Here, only the topology from the ML tree is selected to guide the discussion (Fig. 3). Phylogenetic trees from MP and BI analyses are available as a Suppl. material 1 (Suppl. material 1: Figs S1, S2).

The phylogenetic tree recovered Gasteracanthinae as a monophyletic group with high nodal support for all analyses (Fig. 3, node 1: MP=99/ML=100/BI=1.00). All nominal species within Gasteracanthinae form a well-supported clade. The Gasteracanthinae clade can be divided into three major clades, consisting of (I) a clade of *A. globulata*, *G. hasselti*, and *M. arcuata* (Fig. 3, node 2: MP=100/ ML=100/ BI=1.00); (II) a clade of five *Gasteracantha* species, including *G. cancriformis*, *G. kuhli*, *G. diardi*, *G. diadesmia*, and two lineages that were morphologically identified as *G. doriae* (Fig. 3, node 5: MP=99/ ML=99/ BI=1.00); and (III) a clade of *Thelacantha* (Fig. 3, node 7: MP=97/ ML=100/ BI=1.00). Clade II forms a sister relationship with clade III (Fig. 3, node 4: MP=97/ ML=100/ BI=1.00), while clade I is a sister to clade II + clade III. The only known new-world *Gasteracantha* species, *G. cancriformis*, is placed in a basal position to the rest of *Gasteracantha*. The broad-abdomen *Gasteracantha*, consisting of *G. diadesmia*, *G. diardi*, and two clades of *G. doriae*, form a monophyletic group (Fig. 3, node 6: MP=100/ ML=100/ BI=1.00). Subclades with deep genetic divergences within nominal taxa are detected in *G. cancriformis*, *M. arcuata*, and *T. brevispina*, whereas two clades of *G. doriae* are recovered with a distant relationship. The *G. doriae* clade D1 consists of specimens from Malaysia and juvenile females from Trat and Surat Thani provinces, Thailand; while the Clade D2 contains individuals from Rayong Province, Thailand.

In addition, polyphyly of *Gasteracantha* is revealed. Phylogenetic analyses nest *G. hasselti* together with *M. arcuata* (Fig. 3, node 3), although their phylogenetic relationship is supported only by the ML analysis. The genetic distance also shows that *G. hasselti* is more closely related to *M. arcuata* than other *Gasteracantha* (Table 3). Therefore, we propose to move *G. hasselti* to the genus *Macracantha* as *Macracantha hasselti* (C. L. Koch, 1837) comb. nov. The supporting evidence is further discussed below.

**Table 3.** Average interspecific uncorrected p-distance (%±S.E.) based on the 675 bp COI gene fragment sequences between species within Gasteracanthinae. Average intraspecific distances within each taxon are marked in bold.

Taxa	1	2	3	4	5	6	7	8	9	10	11	12	13	14	15
1. *Actinacantha globulata*	**N/A**														
2. *Gasteracantha cancriformis* (C1)	15.43±1.46	**1.00**±**0.40**													
3. *Gasteracantha cancriformis* (C2)	14.76±1.40	4.26±0.79	**N/A**												
4. *Gasteracantha diadesmia*	14.08±1.35	10.85±1.24	9.84±1.14	**0.84**±**0.25**											
5. *Gasteracantha diardi*	14.48±1.36	11.41±1.30	10.12±1.20	3.77±0.70	**0.31**±**0.14**										
6. *Gasteracantha doriae* (D1)	13.03±1.29	11.28±1.25	10.19±1.16	5.13±0.79	5.40±0.82	**0.45**±**0.21**									
7. *Gasteracantha doriae* (D2)	13.55±1.34	11.65±1.27	10.43±1.17	4.48±0.76	5.15±0.85	5.53±0.82	**0**								
8. *Gasteracantha kuhli*	14.05±1.38	8.68±1.11	8.19±1.02	7.75±0.96	8.36±1.03	7.89±0.99	7.73±0.99	**0.58**±**0.19**							
9. *Macracantha arcuata* (M1)	10.72±1.14	16.49±1.49	15.59±1.40	15.28±1.36	16.45±1.40	15.09±1.34	16.00±1.41	16.12±1.43	**1.30**±**0.29**						
10. *Macracantha arcuata* (M2)	9.64±1.11	15.96±1.50	15.21±1.34	14.19±1.32	15.58±1.38	14.01±1.31	15.06±1.39	13.68±1.32	7.02±0.95	**0.60**±**0.30**					
11. *Macracantha hasselti*	8.33±1.04	14.13±1.39	14.05±1.30	13.06±1.26	13.69±1.30	12.56±1.26	12.81±1.26	12.83±1.26	9.46±1.06	9.21±1.07	**0.72**±**0.21**				
12. *Thelacantha brevispina* (T1)	14.95±1.40	12.91±1.31	12.03±1.22	12.30±1.23	13.29±1.30	12.43±1.26	12.26±1.25	11.86±1.23	15.73±1.37	15.47±1.40	14.08±1.30	**0.17**±**0.12**			
13. *Thelacantha brevispina* (T2)	14.91±1.40	11.53±1.28	10.77±1.20	11.48±1.18	12.05±1.22	11.90±1.24	11.30±1.20	10.56±1.15	16.20±1.38	15.81±1.36	13.58±1.29	5.69±0.91	**0.30**±**0.15**		
14. *Thelacantha brevispina* (T3)	15.12±1.43	13.71±1.38	13.29±1.33	10.78±1.17	11.57±1.22	11.16±1.22	10.86±1.20	11.12±1.21	15.12±1.37	14.21±1.32	13.25±1.29	8.92±1.10	8.19±1.09	**0.15**±**0.15**	
15. *Thelacantha brevispina* (T4)	15.81±1.47	13.77±1.37	12.35±1.25	12.27±1.27	12.63±1.28	13.33±1.31	12.35±1.28	11.64±1.24	14.70±1.37	13.86±1.36	15.06±1.36	9.60±1.15	9.41±1.14	10.25±1.21	**N/A**

Genetic distances of COI gene ranged from 3.77 to 16.49% (average = 10.89%) between taxa, and from 0.15 to 1.30% (average = 0.53%) within taxa (Table 3).

### Species delimitation

**Figure 4.**
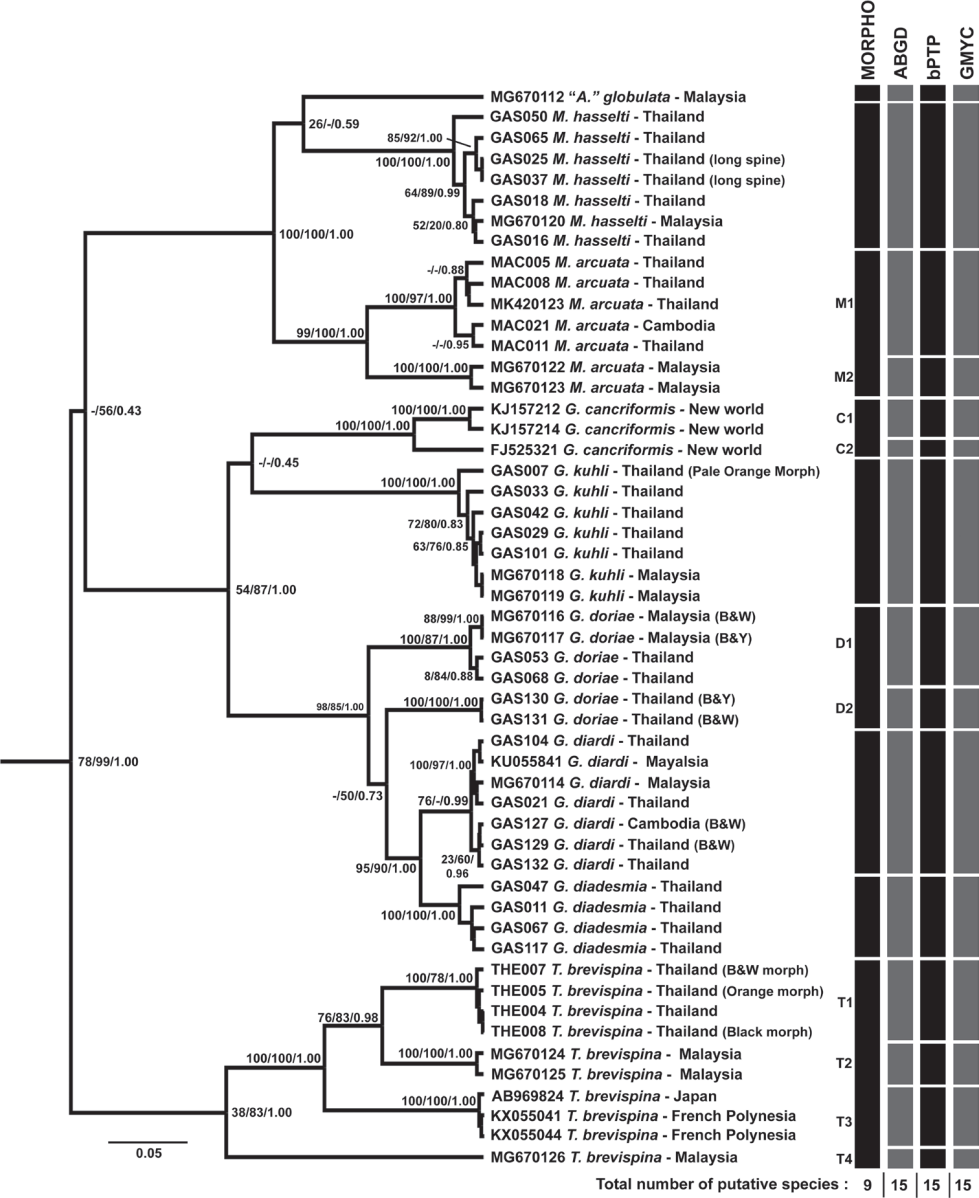
Ultrametric tree generated by BEAST from 675 bp of COI gene showing clusters of OTUs as suggested by morphological identification, and three molecular species delimitation algorithms, ABGD, bPTP, and GMYC. Nodal support values are labeled as MP Jackknife support/ML bootstrap values/Bayesian posterior probability. Gene tree from MP and ML analyses are available as Suppl. material 1 (Suppl. material 1: Figs S3, S4).

All three statistical approaches based on the COI gene dataset generated congruent results for 15 OTUs, corresponding to nine nominal species and six possible cryptic species (Fig. 4). These cryptic species were detected within nominal species: one lineage each in *G. cancriformis*, *G. doriae*, and *M. arcuata*, and three lineages in *T. brevispina*. The delimitation based on the 16S gene generated 14 OTUs for ABGD, 15 OTUs for bPTP, and 10 OTUs for GMYC (Suppl. material 1: Fig. S5). The delimitation based on the H3 gene generated ten OTUs for ABGD, nine OTUs for bPTP, and six OTUs for GMYC (Suppl. material 1: Fig. S6). The delimitation results from the COI dataset were the most consistent with the morphological identification; also, 16S and H3 sequences of some individuals were unavailable for this study. Therefore, only the results from the COI dataset are used for the discussion.

### Diversity of Gasteracanthinae in Thailand

In summary, seven species from three genera, which are *Gasteracantha*, *Macracantha*, and *Thelacantha*, were collected in this study. They are *G. diadesmia*, *G. diardi*, *G. doriae*, *G. kuhli*, *M. arcuata*, *M. hasselti*, and *T. brevispina*. Four other species previously recorded from Thailand, *G. clavigera*, *G. frontata*, *G. irradiata*, and *G. rubrospinis*, were not found during surveys. Therefore, there are eleven named species of Gasteracanthinae present in Thailand including those from previous historical records.

**Order Araneae Clerck, 1757**

**Family Araneidae Clerck, 1757**

**Subfamily Gasteracanthinae Scharff & Coddington, 1997**

### Key to species of spiny-backed orb-weaving spiders subfamily Gasteracanthinae in Thailand

Only species for which specimens were available in this study are included.

**Table:** 

1	Ventral tubercle present. Anterior margin of abdomen forming slight arch between anterior spines. Spinnerets encircled by black sclerotized ring. Spermathecae round or oval	**2**
–	Ventral tubercle absent. Anterior margin of abdomen forming strong arch between anterior spines. Spinnerets placed on elevated black sclerotized structure. Shape of spermathecae not as above	**6**
2	Abdomen much wider than long. Median spines different from other spines. Large trapezoid-shaped sigilla present	**3**
–	Abdomen slightly wider than long. Each pair of spines quite similar in shape. Large trapezoid-shaped sigilla absent	**5**
3	Median spine very large, long, covered with hairs, and arched posteriorly with few marginal spikes. Median sigilla with two small sigilla beside the large trapezoid-shaped sigilla	***G. diardi***
–	Median spine large, with scattered hairs, not arched or slightly arched posteriorly with conspicuous marginal spikes. Median sigilla without two small sigilla beside the large trapezoid-shaped sigilla	**4**
4	Median spine large, thick, plate-like, and directed horizontally. The angle between anterior and posterior spines narrow. Two dark horizontal bands on abdomen straight	***G. diadesmia***
–	Median spines long, thin, less conical, and slightly arched backward. The angle between anterior and posterior spines relatively obtuse. Two dark horizontal bands on abdomen sinuous	***G. doriae***
5	Abdominal spines conical, the bases of anterior and median spines fused. Dorsal abdomen with black and white patches, usually arranged in inverse Y-band. Sternal band hoof-shaped	***G. kuhli***
–	Abdominal spines tubercle-shaped with small projection at the tip. Abdomen various in color. Two large white spots usually present on dorsal abdomen. Sternal band not as above	***T. brevispina***
6	Anterior and posterior spines poorly developed. Median spines very long, at least three times the width of abdomen, slender, and strongly arched	***M. arcuata***
–	Anterior and posterior spines well developed, sharp. Median spine straight, longest, but less than two times the width of abdomen, thick at the base and tapering toward the tip	***M. hasselti***

### Taxonomic account

#### Genus Gasteracantha

Taxon classificationAnimaliaAraneaeAraneidae

Sundevall, 1833

210C35BE-52EE-555A-A781-6D009E2AE9D3

##### Type species.

*Aranea cancriformis* Linnaeus, 1758.

##### Diagnosis.

Cephalic region highly elevated near the middle, abruptly sloped downward posteriorly. Median ocular quadrangle wider behind than in front. Cephalothorax overlapping anterior abdomen. Sternum heart-shaped, pointed posteriorly, concave anteriorly below labium. Abdomen wider than long, with prominent coloration, three pairs of spines, and sigilla on dorsal and ventral sides. Four median sigilla arranged in trapezoid. Dorsal sigilla in three rows, situated near the anterior edge, posterior edge, and behind the posterior edge. Spinnerets encircled by a black sclerotized ring. IV femora elongated.

##### Remarks.

The genus *Gasteracantha* was first described by Sundevall (1833), and subsequently was revised by many authors (Pickard-Cambridge 1879; Dahl 1914; Benoit 1962, 1964; Emerit 1974; Barrion and Litsinger 1995; Levi 1996). Currently, *Gasteracantha* comprises 88 valid species worldwide (World Spider Catalog 2020).

#### Gasteracantha diadesmia

Taxon classificationAnimaliaAraneaeAraneidae

Thorell, 1887

8DAC9447-6FC7-5F68-AC4A-73169908ACAE

Figures 5 , 11A–C

Gasteracantha diadesmia Thorell, 1887: 225. Type locality: Myanmar, Bhamo.
Full list of synonyms and usage of the name available in World Spider Catalog (2020).

##### Material.

Thailand • 3 ♀; Nakhon Ratchasima Province, Wang Nam Khiao District; 14°32.57'N, 101°58.22'E; MUMNH-ARA-GAS009 • 3 ♀; Nakhon Ratchasima Province, Pak Chong District, Phaya Yen; 14°36.97'N, 101°15.90'E; MUMNH-ARA-GAS011 • 2 ♀; Satun Province, Thung Wa District, Khantiphon Cave; 07°05.08'N, 99°47.92'E; MUMNH-ARA-GAS012 • 3 ♀; Ratchaburi Province, Suan Phueng District; 13°33.03'N, 99°17.14'E; MUMNH-ARA-GAS028; MUMNH-ARA-GAS030 • 1 ♀; Kanchanaburi Province, Sai Yok District; 14°24.93'N, 98°52.54'E • 5 ♀; Ratchaburi Province, Suan Phueng District, Pachi Stream; 13°31.18'N, 99°18.88'E; MUMNH-ARA-GAS031 • 1 ♀; Chiang Mai Province, Mueang District; 18°47.00'N, 98°57.13'E; MUMNH-ARA-GAS034 • 3 ♀; Krabi Province, Mueang District, Krabi Noi; 08°07.54'N, 98°55.40'E; MUMNH-ARA-GAS043 • 1 ♀; Bangkok Province, Ratchathewi District, Santiphap Park; 13°45.68'N, 100°32.42'E; MUMNH-ARA-GAS045 • 4 ♀; Mae Hong Son Province, Mueang District, Pang Mu; 19°18.12'N, 97°57.58'E; MUMNH-ARA-GAS047 • 1 ♀ juvenile; Surat Thani Province, Khiri Rat Nikhom District, Wang Badarn Cave; 08°54.52'N, 98°57.08'E; MUMNH-ARA-GAS067 • 2 ♀; Chanthaburi Province, Soi Dao District; 13°06.67'N, 102°12.30'E; MUMNH-ARA-GAS076 • 2 ♀; Chanthaburi Province, Mueang District, Khlong Narai; 12°35.45'N, 102°09.48'E; MUMNH-ARA-GAS077 • 4 ♀; Kanchanaburi Province, Si Sawat District, Na Suan, Ong-ju Canal; 14°48.45'N, 99°05.53'E; MUMNH-ARA-GAS082 • 1 ♀; Phetchabun Province, Lom Sak District; 16°43.74'N, 101°20.22'E; MUMNH-ARA-GAS086 • 2 ♀; Chaiyaphum Province, Phakdi Chumphon District, Ban Chiang, Wua Daeng Cave; 16°04.55'N, 101°26.46'E; MUMNH-ARA-GAS096 • 1 ♀; Loei Province, Nong Hin District; 17°02.41'N, 101°44.18'E; MUMNH-ARA-GAS099 • 2 ♀; Chiang Mai Province, Mae Rim District; 18°55.10'N, 98°54.51'E; MUMNH-ARA-GAS102 • 2 ♀; Kanchanaburi Province, Mueang District, Li Chia Cave; 15°04.50'N, 98°33.96'E; MUMNH-ARA-GAS107 • 3 ♀; Kanchanaburi Province, Thong Pha Phum District, Huai Kayeng; 14°37.85'N, 98°34.32'E; MUMNH-ARA-GAS108 • 5 ♀; Loei Province, Phu Ruea District; 17°31.55'N, 101°15.33'E; MUMNH-ARA-GAS117 • 3 ♀; Chiang Mai Province, Fang District; 19°57.46'N, 99°12.17'E; MUMNH-ARA-GAS122.

##### Diagnosis.

Sternum dark brown with median yellow spot. Abdomen much wider than long. Dorsal side of abdomen with three yellow abdominal horizontal bands: first band on anterior edge near base of anterior spines, second band running between median spines, and third band behind middle sigilla reaching posterior edge. Edge of abdomen with serrated spikes, obvious on spines. Spines dark brown to orange. Anterior spines smallest, obliquely directed. Median spines longest, thick, plate-like, horizontally pointed. Posterior spines conical, pointed backward. Two median yellow spots between the bases of posterior spines. Ventral side of abdomen blackish with scattered yellow spots and small black granules. Ten anterior edge sigilla in total: four sigilla in the middle small, forming a straight line, three sigilla on each side larger, trapezoid. Four median sigilla arranged in trapezoid. Ten posterior edge sigilla in total: six sigilla in the middle, forming a straight line, the pair in the middle close together; two sigilla on each side larger, trapezoid. Five outer posterior edge sigilla, placed near posterior spines. Epigynum subtriangular with two lateral dark patches (Fig. 11A). Scape large, pointed posteriorly, divided into three curves (Fig. 11A, C). Spermathecae round (Fig. 11A), ventrally partially overlapped by wing-shaped sclerotized structure (Fig. 11B). Copulatory ducts encapsulated by sclerotized structure (Fig. 11A). Fertilization ducts emerging posteriorly from spermathecae (Fig. 11A).

##### Variation.

**Figure 5.**
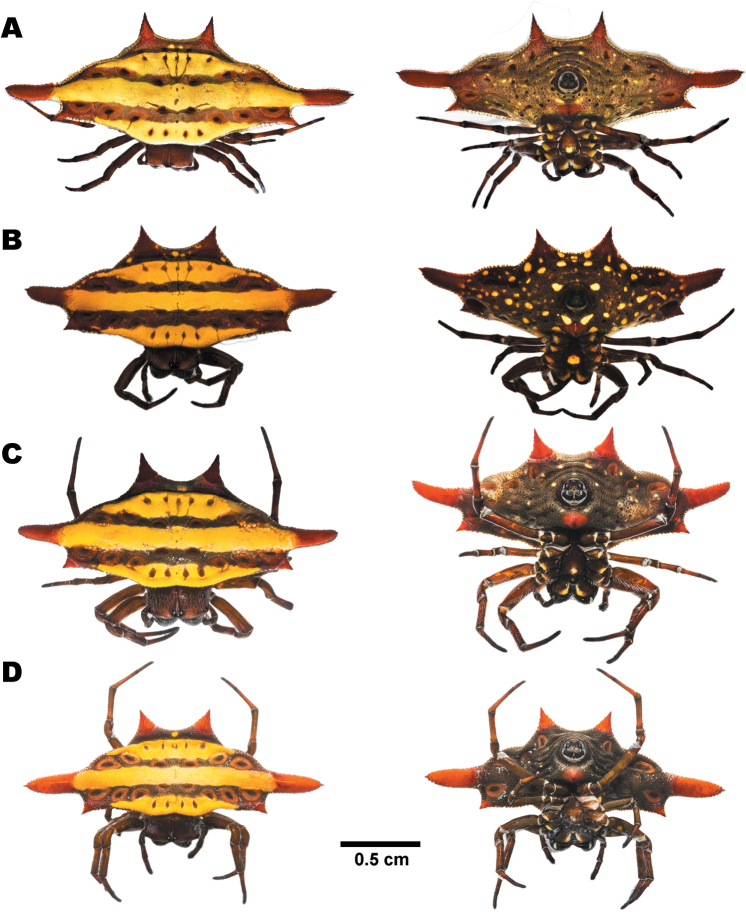
Females of *Gasteracantha diadesmia* showing dorsal view (left) and ventral view (right) **A** specimen from Mae Hong Son (MUMNH-ARA-GAS047) **B** specimen from Nakhon Ratchasima (MUMNH-ARA-GAS011) **C** specimen from Loei (MUMNH-ARA-GAS117) **D** specimen from Chanthaburi (MUMNH-ARA-GAS076).

Dorsal dark horizontal bands, spines, and ventral abdomen either reddish (Fig. 5A, D) or blackish (Fig. 5B, C) in some specimens. Median spines in some specimens slightly pointed backwards (Fig. 5C).

##### Remarks.

*Gasteracantha diadesmia* resembles *Gasteracantha sturi* (Doleschall, 1857), but black horizontal bands of *G. diadesmia* are wider than in *G. sturi* (Doleschall 1857; Kolosváry 1931). Moreover, median spines of *G. diadesmia* are large, thick, and plate-like, while median spines of *G. sturi* are very blunt. *Gasteracantha diadesmia* is distinguished from other Thai species by having broader anterior yellow horizontal band and thick, plate-like, and horizontally pointed median spines. Barrion and Litsinger (1995) reported another form with discontinuous dark horizontal bands from the Philippines. However, this morphotype might belong to another species because the shape of spines and color pattern are different.

##### Distribution and habitat.

India, Myanmar, China, Thailand, Vietnam, Philippines, and Andaman and Nicobar Islands (Yin et al. 1997; World Spider Catalog 2020). *Gasteracantha diadesmia* are commonly found in mixed deciduous forest and dipterocarp forest. The spiders usually construct a vertical web between shrubs in open areas, and sit at the center of the web.

#### Gasteracantha diardi

Taxon classificationAnimaliaAraneaeAraneidae

(Lucas, 1835)

856AC409-5DD3-5AA4-BB66-50F66DAA44BF

Figures 6 , 11D–F

Epeira diardi Lucas, 1835: 70, pl.149, fig. 4. Type locality: Indonesia, Java.
Full list of synonyms and usage of the name available in World Spider Catalog (2020).

##### Material.

Thailand • 3 ♀; Chumphon Province, Sawi District, Wisai Tai; 10°22.38'N, 99°03.61'E; MUMNH-ARA-GAS021 • 3 ♀; Nakhon Ratchasima Province, Pak Chong District, Phaya Yen; 14°36.97'N, 101°15.90'E; MUMNH-ARA-GAS022 • 1 ♀; Nakhon Si Thammarat Province, Chang Klang District; 08°19.27'N, 99°35.39'E; MUMNH-ARA-GAS104 • 4 ♀; Nakhon Si Thammarat Province, Phra Phrom District; 08°22.59'N, 099°52.72'E; MUMNH-ARA-GAS105 • 1 ♀; Chiang Mai Province, Mae Chaem District; 18°28.81'N, 98°22.96'E; MUMNH-ARA-GAS129 • 4 ♀; Nakhon Ratchasima Province, Pak Chong District, Phaya Yen; 14°36.97'N, 101°15.90'E; MUMNH-ARA-GAS132. Cambodia • 3 ♀; Kampot Province; 10°34.92'N, 104°07.21'E; MUMNH-ARA-GAS127.

##### Diagnosis.

Sternum dark brown with small median yellow spot. Abdomen much wider than long. Dorsal side of abdomen dark brown. Edge of abdomen with few serrated spikes. Spines dark brown to orange. Anterior spines smallest, slightly directed obliquely. Median spines very large, covered with hairs, and arched backward. Posterior spines conical, pointed backward. Ventral side of abdomen dark brown with scattered yellow spots and small black granules. Ten anterior edge sigilla in total: four sigilla in the middle smaller, forming a straight line, three sigilla on each side larger, trapezoid. Four median sigilla arranged in a trapezoid, with two small sigilla situated on both lateral sides. Posterior edge with ten sigilla in total: six sigilla in the middle forming a straight line, the pair in the middle closely placed; two sigilla on each side larger, trapezoid. Outer posterior edge with five sigilla near posterior spines. Epigynum subtriangular with two lateral dark patches (Fig. 11D). Scape trapezoid, pointed posteriorly (Fig. 11F). Spermathecae round (Fig. 11D), ventrally partially overlapped by wing-shaped sclerotized structure (Fig. 11E). Copulatory ducts encapsulated by sclerotized structure (Fig. 11D). Fertilization ducts emerging posteriorly from spermathecae (Fig. 11D).

##### Variation.

**Figure 6.**
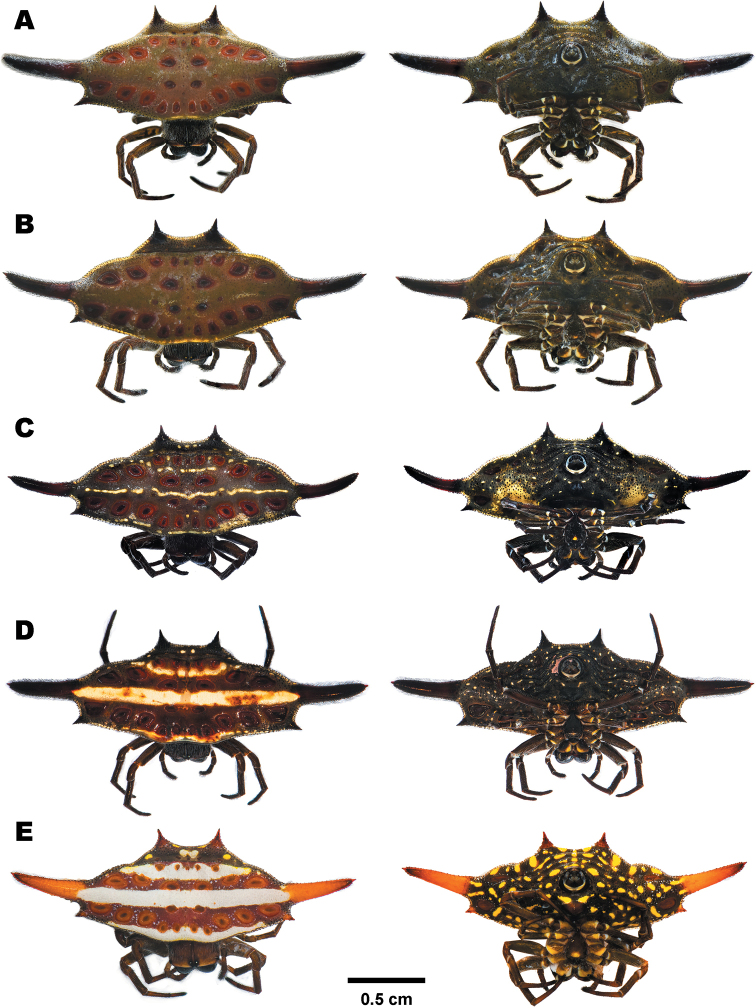
Females of *Gasteracantha diardi* showing dorsal view (left) and ventral view (right) **A, B** dark brown morph **A** specimen from Nakhon Si Thammarat (MUMNH-ARA-GAS104) **B** specimen from Nakhon Si Thammarat (MUMNH-ARA-GAS105) **C** dark red with stripes morph, specimen from Nakhon Ratchasima (MUMNH-ARA-GAS132) **D** narrow horizontal band morph, specimen from Chiang Mai (MUMNH-ARA-GAS129) **E** broad horizontal band morph, specimen from Cambodia, Kampot (MUMNH-ARA-GAS127).

Four morphotypes were found in this study: (1) the dark brown morph is the most common in Thailand. The dorsal abdomen is plain dark brown (Fig. 6A, B). This form is concordant with the description by Lucas (1835). (2) A dark red with stripes morph bears three thin yellow stripes near the anterior margin, between median spines, and in front of posterior sigilla (Fig. 6C). (3) A narrow horizontally banded morph bears three white and three black horizontal lines on dorsal abdomen (Fig. 6D). The first and the third white bands are very narrow. (4) A broad horizontally banded morph possesses three white and two black horizontal lines on dorsal abdomen (Fig. 6E). The median spines are bright orange. Ventral side of abdomen is decorated by bright yellow spots.

##### Remarks.

*Gasteracantha diardi* can be distinguished from other broad-abdomen *Gasteracantha* by its large and posteriorly arched median spines, and two additional small sigilla beside the median trapezoid-shaped sigilla. The original description of *G. diardi* describes the plain dark brown morph specimens (Lucas 1835). In this study, we report three additional color morphs other than the original description. These color morphs are confirmed by molecular phylogenetic analysis in this study (Fig. 4).

##### Distribution and habitat.

India, China, Laos, Thailand, Malaysia, and Indonesia (Java, Borneo, and Sumatra) (Butler 1873; Pickard-Cambridge 1879; Dahl 1914; World Spider Catalog 2020). *Gasteracantha diardi* usually constructs a vertical web between trees, at a height of approximately 2 meters above ground in open areas, and sits at the center of the web.

#### Gasteracantha doriae

Taxon classificationAnimaliaAraneaeAraneidae

Simon, 1877

E7981B0A-6054-5193-BCB0-4EC51FB16855

Figures 7 , 11G–I

Gasteracantha doriae Simon, 1877: 232, pl.3, fig. 3. Type locality: Sarawak, Borneo Island.
Full list of synonyms and usage of the name available in World Spider Catalog (2020).

##### Material.

Thailand • 3 ♀ juvenile; Trat Province, Laem Ngop District; 12°10.39'N, 102°24.33'E; MUMNH-ARA-GAS053 • 1 ♀ juvenile; Surat Thani Province, Khiri Rat Nikhom District, Wang Badarn Cave; 08°54.52'N, 98°57.08'E; MUMNH-ARA-GAS068 • 5 ♀; Rayong Province, Wang Chan District, Pa Yup Nai; 13°01.27'N, 101°26.83'E; MUMNH-ARA-GAS130, MUMNH-ARA-GAS131.

##### Diagnosis.

Sternum brownish black with large yellow spot at the center. Abdomen much wider than long. Dorsal side of abdomen with two black and three white horizontal bands. Two black abdominal horizontal bands arched with sinuous margins. First black horizontal band slightly hollow at the anterior middle. Edge of abdomen with serrated spikes, obvious on spines. Anterior spines smallest, directed obliquely. Posterior spines conical, pointed backward. Median spines longest, less conical, and slightly arched backward. One large median spot between the bases of posterior spines, and one lateral spot on each side. Ventral side of abdomen blackish with chalk-white spots and small black granules. Sigilla reddish brown. Anterior edge with ten sigilla: four sigilla in the middle smaller, forming a straight line, three sigilla on each side larger, trapezoid-shaped. Four median sigilla arranged in trapezoid shape. Posterior edge with ten sigilla: six sigilla in the middle smaller, forming a straight line, with the pair in the middle close together; two sigilla on each side larger, trapezoid. Outer posterior edge with five sigilla near posterior spines. Epigynum with a pair of hook-shaped sclerotized structures between spermathecae, visible in posterior view (Fig. 11I). Spermathecae round (Fig. 11G), ventrally partially overlapped by wing-shaped sclerotized structure (Fig. 11H). Scape long, pointed posteriorly, flanked by lateral sclerotized plates (Fig. 11I). Copulatory ducts encapsulated by sclerotized structure (Fig. 11G). Fertilization duct emerging posteriorly from spermathecae (Fig. 11G).

##### Variation.

**Figure 7.**
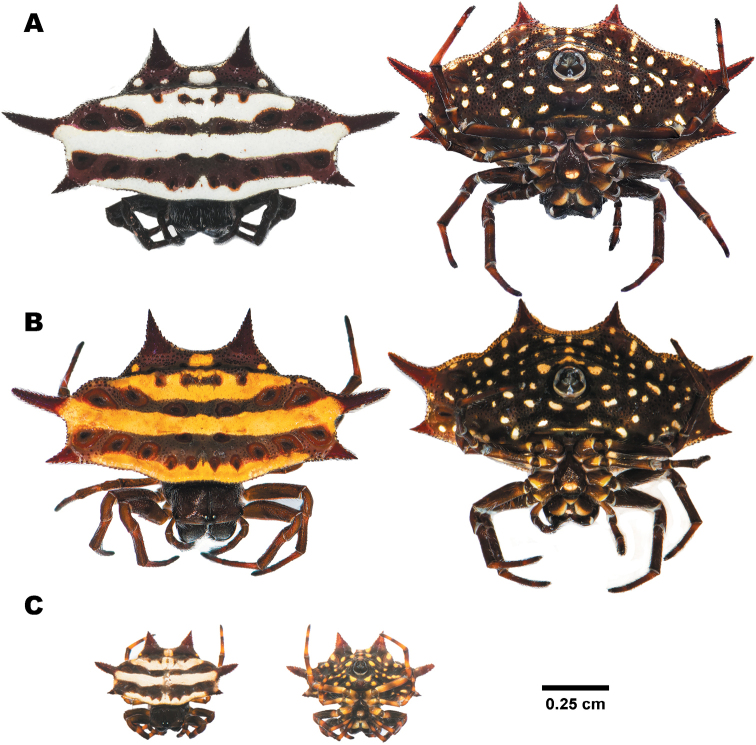
Females of *Gasteracantha doriae***A** black-white bands morph, specimen from Rayong (MUMNH-ARA-GAS131) **B** black-yellow bands morph, specimen from Rayong (MUMNH-ARA-GAS130-1) **C** juvenile, specimen from Trat (MUMNH-ARA-GAS053) **A, B** belong to clade D2 and **C** from clade D1 in Fig. 3.

Two color morphs are observed consisting of the black-white banded morph (Fig. 7A) and the black-yellow banded morph (Fig. 7B). The black bands in the B&Y morph are less sinuous than in the B&W morph.

##### Remarks.

This species resembles *G. frontata*, *G. diadesmia*, and *G. sturi*. These species can be distinguished from each other by abdominal spines and abdominal color pattern. The median spines of *G. doriae* are longer and less conical than *G. frontata*. The median spines of *G. diadesmia* are thicker and wider than *G. doriae*. *Gasteracantha doriae* differs from *G. sturi* in having longer and pointed median spines and wider black horizontal bands. Additionally, the angle between anterior and median spines of *G. doriae* is more obtuse than other species. Although the type specimen of *G. frontata* is without horizontal bands (Blackwall 1864; Pickard-Cambridge 1879), there are some reports stating that *G. frontata* contains abdominal horizontal bands (Pickard-Cambridge 1879; Pocock 1900). Pocock (1900) reported that the first horizontal band of *G. frontata* reaches the base of the anterior spine, whereas the first horizontal band of *G. doriae* terminates before the base of the anterior spine.

Two *Gasteracantha* species with abdominal horizontal bands that were previously recognized as *G. diardi* by Tan et al. (2019) are grouped separately from other Thai *G. diardi* with high nodal support. In addition, these two individuals are morphologically different from other *G. diardi* specimens from Thailand by having smaller size of median spines, as well as different color pattern (horizontal bands morph). By comparing photographs in Tan et al. (2019) and previous taxonomic publications (Simon 1877; Workman and Workman 1892), we propose that these two individuals were *Gasteracantha doriae*. Unfortunately, our specimens (Fig. 7C) in D1 clade that were placed in the same clade with *G. doriae* s.s. Tan et al. (2019) were still juvenile, and therefore we were unable to examine the genitalia.

Interestingly, the phylogenetic tree and species delimitation results suggest another distinct clade in *G. doriae* (clade D2 in Figs 3 and 4). These two clades of *G. doriae* show a distant relationship and potentially are cryptic species. Only a couple of morphological differences can be detected. Morphological characters of *G. doriae* D1, which we observed via photographs in Tan et al. (2019), is similar to the original description (Simon 1877), while *G. doriae* D2 shows morphological variation. The horizontal black bands of *G. doriae* D1 are rather straight with smooth margin, whereas the horizontal black bands of *G. doriae* D2 are curved and with apparently sinuous margin (Figs 7A, B). In addition, *G. doriae* D1 possesses three horizontal black bands, while *G. doriae* D2 presents only two horizontal black bands. The angle between anterior and median spines of *G. doriae* D2 is more obtuse than in *G. doriae* D1. All molecular analyses (i.e., phylogenetic analyses, species delimitation, and genetic distance) in this study strongly suggest that the two lineages are distinct species. However, due to unavailability of adult specimens of *G. doriae* D1, we were unable to compare the female genitalia structure between *G. doriae* D1 and D2, which is usually used as a reliable and distinguishable character in *Gasteracantha* species. Further investigation of adult female specimens from the type locality is necessary to resolve this taxonomic problem.

##### Distribution and habitat.

Indonesia (Borneo), Malaysia, and Thailand (World Spider Catalog 2020). Adult spiders were collected from shrubs and trees. The female spider builds a vertical web between shrubs or trees in open areas. They sit at the center of the web with head directed downward.

#### Gasteracantha kuhli

Taxon classificationAnimaliaAraneaeAraneidae

C. L. Koch, 1837

947E6F43-14D2-58D1-AAC1-A3B446CC8688

Figures 8 , 11J–L

Gasteracantha kuhli C. L. Koch, 1837: 20, fig. 262. Type locality: Indonesia, Java.
Full list of synonyms and usage of the name available in World Spider Catalog (2020).

##### Material.

Thailand • 5 ♀; Nakhon Ratchasima Province, Pak Chong District; 14°31.10'N, 101°24.00'E; MUMNH-ARA-GAS002 • 5 ♀; Ubon Ratchathani Province, Na Chaluai District, Dom Yai Canal; 14°41.25'N, 105°09.27'E; MUMNH-ARA-GAS003 • 2 ♀; Kanchanaburi Province, Si Sawat District; 14°26.49'N, 99°08.06'E; MUMNH-ARA-GAS004 • 2 ♀; Surat Thani Province, Ko Pha-ngan District, Koh Tao Is.; 10°04.04'N, 99°49.10'E; MUMNH-ARA-GAS005 • 5 ♀; Samut Prakan Province, Phra Pradaeng District, Bang Kachao; 13°41.50'N, 100°33.46'E; MUMNH-ARA-GAS005 • 1 ♀; Surat Thani Province, Phanom District, Khlong Sok; 08°54.20'N, 98°31.81'E; MUMNH-ARA-GAS007 • 4 ♀; Kanchanaburi Province, Sai Yok District; 14°24.93'N, 98°52.54'E; MUMNH-ARA-GAS027 • 5 ♀; Ratchaburi Province, Suan Phueng District; 13°33.03'N, 99°17.14'E; MUMNH-ARA-GAS029 • 5 ♀; Ratchaburi Province, Suan Phueng District, Pachi Stream; 13°31.18'N, 99°18.88'E; MUMNH-ARA-GAS030 • 5 ♀; Samut Prakan Province, Phra Pradaeng District, Bang Kachao; 13°41.85'N, 100°33.93'E; MUMNH-ARA-GAS033 • 4 ♀; Trang Province, Mueang District, Ban Pho; 07°34.18'N, 99°39.16'E; MUMNH-ARA-GAS039 • 2 ♀; Ranong Province, Suk Samran District, Na Kha; 09°23.75'N, 98°25.75'E; MUMNH-ARA-GAS040 • 5 ♀; Krabi Province, Mueang District, Krabi Noi; 08°07.54'N, 98°55.50'E; MUMNH-ARA-GAS042 • 5 ♀; Phrae Province, Mueang District; 18°08.58'N, 100°07.81'E; MUMNH-ARA-GAS046 • 4 ♀; Mae Hong Son Province, Mueang District, Pang Mu; 19°18.12'N, 97°57.58'E; MUMNH-ARA-GAS048 • 2 ♀; Mae Hong Son Province, Mae La Noi District, Mae La Luang; 18°32.31'N, 97°53.83'E; MUMNH-ARA-GAS049 • 1 ♀; Loei Province, Mueang District, Kok Thong; 17°30.53'N, 101°35.83'E; MUMNH-ARA-GAS054 • 1 ♀; Loei Province, Pak Chom District, Huai Bo Suen; 17°44.78'N, 101°58.33'E; MUMNH-ARA-GAS055 • 5 ♀; Udon Thani Province, Nam Som District; 17°46.93'N, 102°06.02'E; MUMNH-ARA-GAS056 • 1 ♀; Nakhon Ratchasima Province, Mueang District, Suranari University; 14°52.97'N, 102°01.27'E; MUMNH-ARA-GAS057 • 5 ♀; Prachuap Khiri Khan Province, Bang Saphan District, Khao Ma Rong Cave; 11°12.17'N, 99°29.65'E; MUMNH-ARA-GAS060 • 2 ♀; Chumphon Province, Tha Sae District, Pisadarn Cave; 10°45.60'N, 99°13.77'E; MUMNH-ARA-GAS063 • 2 ♀; Surat Thani Province, Khiri Rat Nikhom District, Wang Badarn Cave; 08°54.52'N, 98°57.83'E; MUMNH-ARA-GAS066 • 1 ♀; Sa Kaeo Province, Khlong Hat District, Phet Pho Thong Cave; 13°24.80'N, 102°19.55'E; MUMNH-ARA-GAS072 • 1 ♀; Lopburi Province, Mueang District, Kok Toom; 14°48.80'N, 100°47.63'E; MUMNH-ARA-GAS074 • 1 ♀; Lopburi Province, Mueang District, Phra Tad Cave; 14°48.40'N, 100°49.48'E; MUMNH-ARA-GAS075 • 1 ♀; Chanthaburi Province, Mueang District, Khlong Narai; 12°35.48'N, 102°09.45'E; MUMNH-ARA-GAS078 • 2 ♀; Nakhon Nayok Province, Pak Phli District, Khun Dan Prakarn Chon Dam; 14°18.88'N, 101°19.27'E; MUMNH-ARA-GAS080 • 4 ♀; Kanchanaburi Province, Si Sawat District, Ong-ju Canal; 14°48.45'N, 99°05.53'E; MUMNH-ARA-GAS084 • 1 ♀; Phetchabun Province, Si Thep District, 15°28.52'N, 100°58.53'E; MUMNH-ARA-GAS085 • 1 ♀; Phetchabun Province, Lom Sak District; 16°43.73'N, 101°20.22'E; MUMNH-ARA-GAS087 • 2 ♀; Loei Province, Wang Saphung District, Pha Bing, 17°14.05'N, 101°45.63'E; MUMNH-ARA-GAS089 • 4 ♀; Phetchabun Province, Wichian Buri District, Wat Tham Thep Bandan, 15°45.42'N, 101°02.27'E; MUMNH-ARA-GAS090 • 2 ♀; Loei Province, Wang Saphung District, Pha Bing; 17°14.47'N, 101°44.25'E; MUMNH-ARA-GAS091 • 2 ♀; Loei Province, Chiang Khan District, Bu Hom; 17°55.05'N, 101°45.13'E; MUMNH-ARA-GAS092 • 1 ♀; Loei Province, Phu Kradueng District, Pha Nok Khao; 16°53.65'N, 101°57.28'E; MUMNH-ARA-GAS093 • 2 ♀; Phetchabun Province, Mueang District, Wat Nam Pang Cave; 16°14.77'N, 101°08.17'E; MUMNH-ARA-GAS094 • 3 ♀; Chaiyaphum Province, Phakdi Chumphon District, Wua Daeng Cave; 16°04.55'N, 101°26.45'E; MUMNH-ARA-GAS095 • 3 ♀; Loei Province, Nong Hin District; 17°02.42'N, 101°44.18'E; MUMNH-ARA-GAS098 • 3 ♀; Chiang Mai Province, Mae Rim District, Mae Raem; 18°55.10'N, 98°54.52'E; MUMNH-ARA-GAS101 • 1 ♀; Chiang Mai Province, Mueang District; 18°46.93'N, 98°57.53'E; MUMNH-ARA-GAS103 • 2 ♀; Kanchanaburi Province, Thong Pha Phum District, Huai Kayeng; 14°37.85'N, 98°34.32'E; MUMNH-ARA-GAS110 • 1 ♀; Ratchaburi Province, Mueang District, Nam Phu; 13°33.47'N, 99°36.97'E; MUMNH-ARA-GAS114 • 2 ♀; Chiang Rai Province, Mae Fa Luang District; 20°14.23'N, 99°49.42'E; MUMNH-ARA-GAS123 • 3 ♀; Chiang Mai Province, Chiang Dao District; MUMNH-ARA-GAS124.

##### Diagnosis.

Sternum black with dull yellow hoof-shaped patch. Abdomen octagonal, slightly wider than long. Dorsal side of abdomen with black and white patches. Edge of abdomen smooth. Three pairs of spines similar in shape. Bases of anterior spines and median spines fused. Ventral side of abdomen blackish brown with scattered chalky yellow stripes. Anterior edge with ten sigilla in total: four sigilla in the middle, three sigilla on each side, placed near the base of anterior spines. Four median sigilla arranged in trapezoid shape. Posterior edge with ten sigilla in total: six sigilla in the middle near posterior margin, forming a straight line, the pair in the middle closely placed. Outer posterior edge with five sigilla, placed near posterior spines. Epigynum subtriangular with small subtriangular scape (Fig. 11F). Spermathecae round (Fig. 11G), ventrally partially overlapped by unconnected sclerotized structures on each side (Fig. 11H). Copulatory ducts encapsulated by sclerotized structure (Fig. 11G). Fertilization ducts emerging posteriorly from spermathecae (Fig. 11G).

##### Variation.

**Figure 8.**
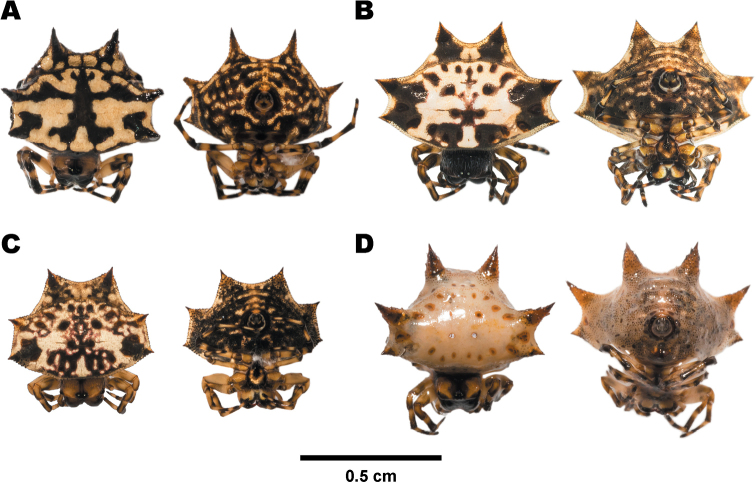
Females of *Gasteracantha kuhli* showing dorsal view (left) and ventral view (right) **A–C** black-white morph **A** specimen from Samut Prakan (MUMNH-ARA-GAS033) **B** specimen from Lopburi (MUMNH-ARA-GAS074) **C** specimen from Ratchaburi (MUMNH-ARA-GAS029) **D** pale orange morph, specimen from Surat Thani (MUMNH-ARA-GAS007).

Color patterns on the abdomen of *G. kuhli* are variable, but commonly with inverse Y-band markings on the dorsal abdomen (Fig. 8A-C). Another morph is pale orange (Fig. 8D). This morph is newly discovered in this study. Its description is as follows: cephalothorax blackish brown with large dull yellow patches on each side, slightly longer than wide, clothed with short white hairs. Cephalic region highly elevated and abruptly sloped downward posteriorly, thoracic region overlapped by anterior side of abdomen. Eight eyes arranged into two rows subequal in size, located above the frontal margin: four median eyes form a trapezoid and are placed on a small protuberance at the middle of frontal margin, lateral eyes on each side placed on a tubercle near corner of frontal margin. Sternum dark brown with large hoof-shaped patch. Abdomen slightly wider than long, pale beige with small brown spots on margin. Six abdominal spines orangish brown, conical, tapering toward the tip. Anterior spines smallest, directed obliquely. Median spines pointed obliquely. Posterior spines largest, pointed backward with small brown spots near the bases. Ventral side of abdomen pale orange with scattered brown granules. Sigilla orangish brown. Ten anterior edge sigilla in total: six sigilla in the middle, two sigilla on each side near the base of anterior spines. Four median sigilla arranged in trapezoid. Ten posterior edge sigilla in total, the pair in the middle placed close together. Outer posterior edge with five sigilla near posterior spines.

##### Distribution and habitat.

Bhutan, China, Japan, Korea, Hong Kong, Taiwan, Cambodia, Thailand, Myanmar, Andaman and Nicobar Islands, Indonesia (Java, and Sumatra), Phi1ippines, and Singapore (Barrion and Litsinger 1995; Sen et al. 2015; World Spider Catalog 2020). *Gasteracantha kuhli* can be found in several habitats such as paddy fields, dipterocarp forest, dry evergreen forest and agriculture areas. The female spider builds a vertical web between shrubs or trees in open areas. The spiders sit at the center of web with head pointed downwards.

#### Gasteracantha clavigera

Taxon classificationAnimaliaAraneaeAraneidae

Giebel, 1863

A863E75D-62FC-5EDF-9173-378F18769A79

Gasteracantha clavigera Giebel, 1863: 307. Type locality: Siam.
Full list of synonyms and usage of the name available in World Spider Catalog (2020).

##### Remarks.

The abdomen of *G. clavigera* is octagonal, slightly wider than long. Color of the abdomen is yellow, with black stripes near the anterior edge. The appearance of this species is similar to *M. hasselti* and *M. arcuata.* However, tips of median spines of *G. clavigera* are club-shaped, and decorated with a tuft of hairs (Giebel 1863; Butler 1873; Simon 1877).

*Gasteracantha clavigera* was described by Giebel (1863). However, only the name “Siam” [= Thailand] was mentioned, without any location details. *Gasteracantha clavigera* has been reported in the Malay Archipelago. Based on its distribution records from previous study, this species might be found in the southern part of Thailand (World Spider Catalog 2020).

##### Distribution.

Thailand, Philippines (Luzon, Manilla, and Samar), and Indonesia (Sulawesi) (Dahl 1914; World Spider Catalog 2020).

#### Gasteracantha frontata

Taxon classificationAnimaliaAraneaeAraneidae

Blackwall, 1864

5D24C7C3-ABD5-5D20-9AD5-574F5A6839E9

Gasteracantha frontata Blackwall, 1864: 40. Type locality: East Indies.
Full list of synonyms and usage of the name available in World Spider Catalog (2020).

##### Remarks.

The abdomen of *G. frontata* is wider than long. Color of the abdomen is brownish yellow. Median spines of *G. frontata* are conical, and not elongated compared to other *Gasteracantha* species with a broad abdomen (Blackwall 1864; Pickard-Cambridge 1879). *Gasteracantha frontata* were reported from Chanthaburi and Rayong provinces (Simon 1886). However, we failed to obtain specimens from either area in this study.

##### Distribution.

East Indies, India, Thailand, Myanmar, Vietnam, and Indonesia (Simon 1886; World Spider Catalog 2020).

#### Gasteracantha irradiata

Taxon classificationAnimaliaAraneaeAraneidae

(Walckenaer, 1841)

CFE50804-D92B-5D66-AFEE-37F72278889D

Plectana irradiata Walckenaer, 1841: 170. Type locality: Cochinchina.
Full list of synonyms and usage of the name available in World Spider Catalog (2020).

##### Remarks.

The abdomen of *G. irradiata* is oval and wider than long. Color of the abdomen is yellowish. The anterior edge of the abdomen is strongly curved backwards. Abdominal sigilla are very small. Abdominal spines are reddish. Anterior spines are shortest. Median spines are longest (Walckenaer 1841; Merian 1911; Dahl 1914).

The specimens of *G. irradiata* collected from Thailand belong to Dahl’s collection (Dahl 1914). However, the sampling locality was only noted as “Siam.” Based on its distribution records from previous study, it is possible that *G. irradiata* might be found in the southern and/or eastern parts of Thailand (World Spider Catalog 2020). We failed to collect *G. irradiata* in this study.

##### Distribution and habitat.

Vietnam, Thailand, and Indonesia (Sulawesi, Sumatra, Lombok, and Java) (Dahl 1914; World Spider Catalog 2020).

#### Gasteracantha rubrospinis

Taxon classificationAnimaliaAraneaeAraneidae

Guérin, 1838

26AE522E-67F0-567B-B027-B3A6C687BF6C

Gasteracantha rubrospinis Guérin, 1838: 53. Type locality: Waigiou [Waigeo Island].
Full list of synonyms and usage of the name available in World Spider Catalog (2020).

##### Remarks.

The abdomen of *Gasteracantha rubrospinis* is wider than long. This species can be distinguished from other Thai *Gasteracantha* by characteristics of their spines and the color pattern on the dorsal abdomen. The abdomen is bright yellow, with a large and incomplete horizontal black transverse band near the anterior edge. The abdominal spines are wider at the base, tapered toward the tip, and ending with a sharp point (Guérin 1838; Simon 1877; Pocock 1897). The reported specimens of *G. rubrospinis* from Thailand belong to Pocock’s collection (Pocock 1897). The locality was listed as “Patani” [= Pattani Province], the southernmost province of Thailand. No specimens were obtained in this study.

##### Distribution and habitat.

Indonesia (Moluccas, Sulawesi, Lombok), New Caledonia, Guam, Thailand (Pattani Province) (Pocock 1897; World Spider Catalog 2020).

#### Genus Macracantha

Taxon classificationAnimaliaAraneaeAraneidae

Simon, 1864

EA5E140D-B608-5462-8220-5FDC0608300C

##### Type species.

*Aranea arcuata* Fabricius, 1793

##### Diagnosis.

Cephalic region highly elevated near the middle, abruptly sloped downward posteriorly. Median ocular quadrangle wider behind than in front. Cephalothorax overlapping anterior abdomen. Sternum heart-shaped, pointed posteriorly, concave anteriorly below labium. Abdomen octagonal with three pairs of spines, and sigilla on dorsal and ventral sides. Anterior edge of abdomen curved between median spines. Dorsal sigilla teardrop-shaped, subequal in size, arranged in three rows, and situated near the anterior edge, posterior edge, and behind the posterior edge. Four median sigilla arranged in a trapezoid. Median spines well developed, elongated. Ventral tubercle is absent. Spinnerets placed on elevated black sclerotized structure, forming a shape like a shield volcano. Legs elongated.

##### Remarks.

*Macracantha* was formerly classified as a subgenus of *Gasteracantha*, but later elevated to an independent genus by Emerit (1974). This genus now consists of two species, *M. arcuata* (World Spider Catalog 2020) and *M. hasselti* (this study). The latter species is currently transferred to *Macracantha* according to phylogenetic analyses and anatomical evidence in this study.

#### Macracantha arcuata

Taxon classificationAnimaliaAraneaeAraneidae

(Fabricius, 1793)

D2B75A31-EC9C-5847-9131-D77FA2240426

**Figure 9.**
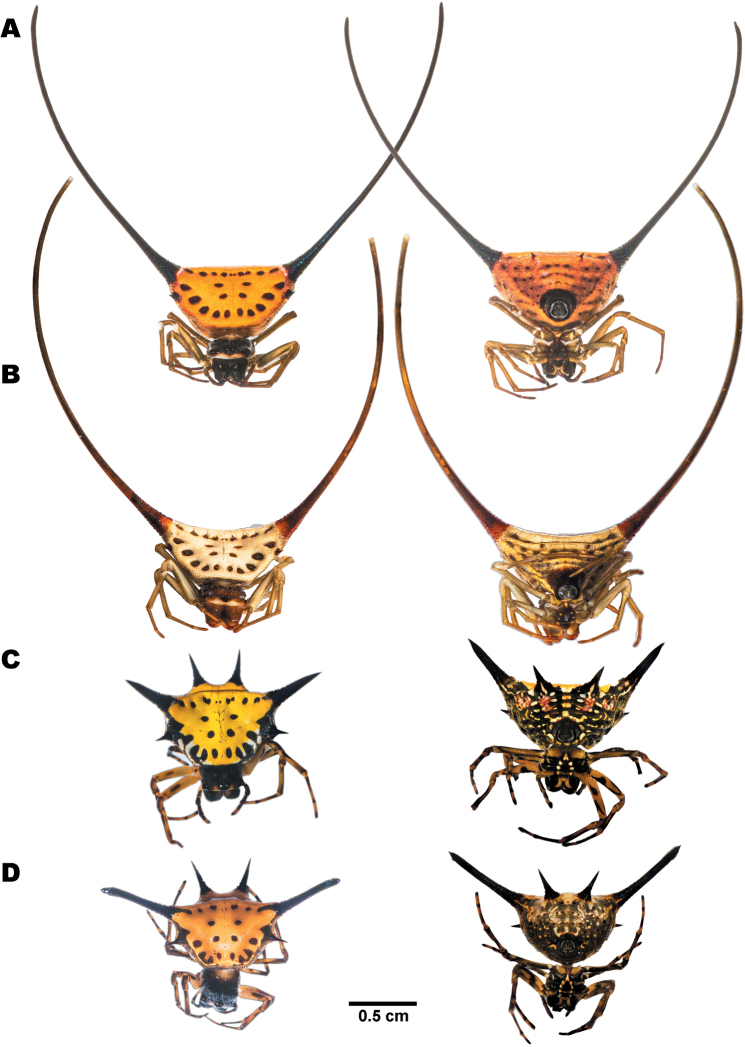
Females of **A, B***Macracantha arcuata* and **C, D***M. hasselti* showing dorsal view (left) and ventral view (right) **A** orange morph, specimen from Prachuap Khiri Khan (MUMNH-ARA-MAC008) **B** white morph, specimen from Kanchanaburi (MUMNH-ARA-MAC013-W1) **C** sharp spines morph, specimen from Saraburi (MUMNH-ARA-GAS018) **D** long spines morph, specimen from Phetchaburi (MUMNH-ARA-GAS025).

Figures 9A, B , 12A–C

Aranea arcuata Fabricius, 1793: 425. Type locality: East Indies.
Full list of synonyms and usage of the name available in World Spider Catalog (2020).

##### Material.

Thailand • 4 ♀; Krabi Province, Mueang District, Krabi Noi; 08°07.45'N, 98°55.45'E; MUMNH-ARA-MAC002 • 1 ♀; Phang-nga Province, Thap Put District, 08°35.58'N, 98°40.08'E; MUMNH-ARA-MAC003 • 2 ♀; Nakhon Ratchasima Province, Pak Chong District; 14°31.10'N, 101°24.00'E; MUMNH-ARA-MAC004 • 5 ♀; Krabi Province, Plai Phraya District, Khao Khao Hua Ling; 08°30.88'N, 98°45.57'E; MUMNH-ARA-MAC005 • 1 ♀; Ranong Province, Mueang District, Hat Som paen; 09°57.55'N, 98°39.57'E; MUMNH-ARA-MAC007 • 4 ♀; Prachuap Khiri Khan Province, Bang Saphan District, Khao Ma Rong Cave; 11°12.17'N, 99°29.65'E; MUMNH-ARA-MAC008 • 2 ♀; Phang-nga Province, Mueang District; 08°26.57'N, 98°30.95'E; MUMNH-ARA-MAC009 • 2 ♀; Phetchabun Province, Lom Sak District; 16°43.74'N, 101°20.22'E; MUMNH-ARA-MAC010 • 2 ♀; Chiang Mai Province, Mae Rim District, Pong Yaeng; 18°53.93'N, 98°51.58'E; MUMNH-ARA-MAC011 • 1 ♀; Nakhon Si Thammarat Province, Chang Klang District; 08°19.27'N, 99°35.38'E; MUMNH-ARA-MAC012 • 4 ♀; Kanchanaburi Province, Sai Yok District; 14°24.93'N, 98°52.53'E; MUMNH-ARA-MAC013 • 1 ♀; Chumphon Province, Mueang District, Ban Na; 10°27.43'N, 99°02.58'E; MUMNH-ARA-MAC015 • 2 ♀; Mae Hong Son Province, Mae Sariang District, Mae Ho, 18°03.78'N, 98°02.20'E; MUMNH-ARA-MAC016 • 2 ♀; Chiang Mai Province, Mae Taeng District; 19°10.71'N, 98°54.95'E; MUMNH-ARA-MAC017 • 3 ♀; Chumphon Province, Sawi District, Nam Lot Cave; 10°14.03'N, 98°56.68'E; MUMNH-ARA-MAC019 • Chumphon Province, Mueang District, Wat Tham Sing; 10°25.58'N, 99°03.63'E; MUMNH-ARA-MAC020 • 1 ♀; Chanthaburi Province, Laem Sing District; 12°31.12'N, 102°10.23'E; MUMNH-ARA-MAC022. Cambodia • 2 ♀; Cambodia Province, Kampot District; 10°34.92'N, 104°07.22'E; MUMNH-ARA-MAC021.

##### Diagnosis.

Sternum black with yellow patches near anterior edge, coxae II and III, and the apex. Abdomen octagonal, orange, and slightly wider than long. Anterior edge of abdomen curved between anterior spines. Median spines very long, slender, and strongly arched, three times the abdomen width. Anterior and posterior spines poorly developed. Ventral side of abdomen orange. Spinnerets placed on strongly elevated black sclerotized structure. Ten anterior edge sigilla subequal in size. Four median sigilla arranged in a trapezoid. Ten posterior edge sigilla arranged in a straight line, closely spaced together. Outer posterior edge with nine sigilla: five sigilla placed near posterior spines, two sigilla on each side. Epigynum wider than long, with transparent median groove, visible in ventral view (Fig. 12B). Scape tongue-shaped, with strongly recurved tip, visible in ventral view (Fig. 12B). Spermathecae reniform (Fig. 12A). Copulatory ducts bulging distally, encapsulated by sclerotized structure (Fig. 12A, C). Fertilization ducts emerging posteriorly from spermathecae (Fig. 12A).

##### Variation.

Two plain color morphs were found in this study, consisting of an orange morph (Fig. 9A), and a white morph (Fig. 9B). The orange morph was the most common, whereas the white morph was found rarely within some populations.

##### Distribution and habitat.

India, Sri Lanka, China, Myanmar, Malaysia, Thailand, Cambodia, and Indonesia (Java and Sumatra) (Tikader 1982; Yin et al. 1997; World Spider Catalog 2020). *Macracantha arcuata* builds a vertical web under the shade of large trees or thick bushes. The female spider hangs at the underside of the web.

#### Macracantha hasselti

Taxon classificationAnimaliaAraneaeAraneidae

(C. L. Koch, 1837) comb. nov.

6D483374-4B4E-50FC-86C5-A821DB4CB422

Figures 9C–D , 12D–I

Gasteracantha hasseltii C. L. Koch, 1837: 29, fig. 267. Type locality: Indonesia, Java.
Full list of synonyms and usage of the name available in World Spider Catalog (2020).

##### Material.

Thailand • 3 ♀; Nakhon Ratchasima Province, Wang Nam Khiao District; 14°32.57'N, 101°58.22'E; MUMNH-ARA-GAS013 • 2 ♀; Ratchaburi Province, Suan Phueng District, 13°34.88'N, 99°10.79'E; MUMNH-ARA-GAS014 • 2 ♀; Nan Province, Tha Wang Pha District; 19°08.45'N, 100°45.38'E; MUMNH-ARA-GAS15 • 5 ♀; Ubon Ratchathani Province, Na Chaluai District, Wat Phupansoong; 14°30.30'N, 105°16.33'E; MUMNH-ARA-GAS016 • 1 ♀; Nakhon Ratchasima Province, Pak Chong District; 14°31.58'N, 101°22.13'E; MUMNH-ARA-GAS017 • 2 ♀; Saraburi Province, Kaeng Khoi District, Tha Maprang; 14°29.85'N, 101°08.25'E; MUMNH-ARA-GAS018 • 4 ♀; Phrae Province, Rong Kwang District, Huai Rong Waterfall; 18°26.51'N, 100°27.01'E; MUMNH-ARA-GAS019 • 5 ♀; Ubon Ratchathani Province, Det Udom District, Non Sombun; 14°47.44'N, 105°06.16'E; MUMNH-ARA-GAS020 • 2 ♀; Nakhon Ratchasima Province, Pak Chong District, Phaya Yen; 14°36.97'N, 101°15.90'E; MUMNH-ARA-GAS024 • 2 ♀; Phetchaburi Province, Kaeng Krachan District; 12°53.41'N, 99°39.32'E; MUMNH-ARA-GAS025 • 1 ♀; Phetchaburi Province, Kaeng Krachan District; 12°54.68'N, 99°38.45'E; MUMNH-ARA-GAS037 • 2 ♀; Chiang Mai Province, Mae Taeng District; 19°08.51'N, 98°54.94'E; MUMNH-ARA-GAS038 • 4 ♀; Mae Hong Son Province, Mueang District, Pang Mu; 19°18.12'N, 097°57.73'E; MUMNH-ARA-GAS049 • 5 ♀; Mae Hong Son Province, Mae La Noi District, Mae La Luang; 18°32.31'N, 97°53.83'E; MUMNH-ARA-GAS050 • 3 ♀; Prachuap Khiri Khan Province, Bang Saphan District, Wat Tham Khao Wong; 11°17.47'N, 99°29.72'E; MUMNH-ARA-GAS062 • 5 ♀; Chumphon Province, Tha Sae District, Pisadarn Cave; 10°45.60'N, 99°13.77'E; MUMNH-ARA-GAS065 • 5 ♀; Sa Kaeo Province, Khlong Hat District, Saeng Tian Cave; 13°18.93'N, 102°19.91'E; MUMNH-ARA-GAS070 • 5 ♀; Sa Kaeo Province, Khao Chakan District, Wat Tham Khao Chan; 13°34.73'N, 102°05.56'E; MUMNH-ARA-GAS073 • 5 ♀; Kanchanaburi Province, Si Sawat District, Ong-ju Canal; 14°48.45'N, 99°05.53'E; MUMNH-ARA-GAS083 • 4 ♀; Phetchabun Province, Lom Sak District; 16°43.74'N, 101°20.22'E; MUMNH-ARA-GAS088 • 3 ♀; Chaiyaphum Province, Phakdi Chumphon District, Wua Daeng Cave; 16°04.55'N, 101°26.46'E; MUMNH-ARA-GAS097 • 3 ♀; Chiang Mai Province, Mae Rim District, Mae Raem; 18°55.10'N, 98°54.51'E; MUMNH-ARA-GAS100 • 3 ♀; Kanchanaburi Province, Thong Pha Phum District, Huai Kayeng; 14°37.85'N, 98°34.32'E; MUMNH-ARA-GAS109 • 1 ♀; Kanchanaburi Province, Sai Yok District, Tha Sao; 14°21.14'N, 98°57.28'E; MUMNH-ARA-GAS113 • 1 ♀; Kanchanaburi Province, Si Sawat District, Khao Chot; 14°48.26'N, 99°10.93'E; MUMNH-ARA-GAS121 • 3 ♀; Kanchanaburi Province, Si Sawat District, Tha Kradan; 14°22.41'N, 99°09.02'E; MUMNH-ARA-GAS125.

##### Diagnosis.

Sternum black with yellow patches near anterior edge, coxae II and III, and the apex. Abdomen octagonal. Anterior edge of abdomen curved between anterior spines. Dorsal side of abdomen yellow with black and white patches near anterior margin. Anterior and posterior spines small, and sharp at the tips. Median spines longest, tapering toward the tip. Ventral side of abdomen black with scattered yellow stripes. Spinnerets placed on strongly elevated black sclerotized structure. Ten anterior edge sigilla subequal in size. Four median sigilla arranged in a trapezoid. Ten posterior edge sigilla arranged in a straight line, with the first pair and the second and third sigilla from the middle close together. Outer posterior edge with nine sigilla in total: five sigilla placed near posterior spines, two sigilla on each side. Epigynum subtriangular with sock-shaped structures, opposite to each other (Fig. 12D, G). Scape very long, tongue-shaped, pointed posteriorly (Fig. 12E, H). Spermathecae balloon-shaped (Fig. 12D, G). Copulatory ducts bulging distally, encapsulated by sclerotized structure (Fig. 12D, G). Fertilization ducts emerging posteriorly from spermathecae (Fig. 12D, G).

##### Variation.

The patch near abdominal anterior margin is narrow or absent in some specimens. Two morphs are found in this study: a sharp spines morph (Figs 9C; 12D–F) with its morphology as in the diagnosis, and a long spines morph (figs 9D; 12G–I), which is characterized by the six abdominal spines being longer than in the sharp spines morph. The median spines are longest, straight without tapering, and with spikes at the bases. The epigynum of the two morphs are similar in shape.

##### Remarks.

*Macracantha hasselti* was once classified in genus *Gasteracantha* (World Spider Catalog 2020). However, the phylogenetic tree in this study recovered a sister relationship between *M. arcuata* and *M. hasselti*, which is supported by their synapomorphic characters (see discussion) in both external and internal morphologies. Based on this evidence, we propose to reclassify these two species in the same genus.

The long spines morph resembles *Gasteracantha dalyi* Pocock, 1900, especially as their female genital structures are identical (Tikader 1982). They are differentiated from each other by the morphology of abdominal spines. Anterior and posterior spines of *M. hasselti* are longer and the median spines are shorter than in *G. dalyi* (Tikader 1982).

##### Distribution and habitat.

India, China, Cambodia, Vietnam, Myanmar, Thailand, Malaysia, Singapore, and Indonesia (Java, and Sumatra) (Yin et al. 2012; Sen et al. 2015; World Spider Catalog 2020). *Macracantha hasselti* builds a vertical web under the shade of large trees or thick shrubs.

#### Genus Thelacantha

Taxon classificationAnimaliaAraneaeAraneidae

Hasselt, 1882

C11A2817-29F2-5991-A733-5198F014E34B

##### Type species.

*Plectana brevispina* Doleschall, 1857.

##### Diagnosis.

Cephalic region highly elevated in middle, abruptly sloping downward posteriorly. Median ocular quadrangle wider behind than in front. Cephalothorax overlapping abdomen. Sternum heart-shaped, pointed posteriorly, and concave anteriorly below labium. Abdomen octagonal, with sigilla on dorsal and ventral sides. Three pairs of abdominal spines, tubercle, with a small protuberance at the tip. Dorsal sigilla in three rows, situated near the anterior edge, posterior edge, and behind the posterior edge. Four median sigilla arranged in a trapezoid. Ventral tubercle is present. Spinnerets encircled by black sclerotized rings.

##### Remarks.

*Thelacantha* was a subgenus of *Gasteracantha*, but later proposed to be a genus (Benoit 1964; Emerit 1974), which is now monotypic (World Spider Catalog 2020).

#### Thelacantha brevispina

Taxon classificationAnimaliaAraneaeAraneidae

(Doleschall, 1857)

3F5E4CDB-0C2A-562B-9CE7-63B195D67126

Figures 10 , 12J–L

Plectana brevispina Doleschall, 1857: 423. Type locality: Indonesia, Ambon Island.
Full list of synonyms and usage of the name available in World Spider Catalog (2020).

##### Material.

Thailand • 4 ♀; Samut Sakhon Province, Khok Kham District; 13°29.27'N, 100°20.13'E; MUMNH-ARA-THE003 • 3 ♀; Phetchaburi Province, Ban Laem District; 13°02.55'N, 100°05.55'E; MUMNH-ARA-THE004 • 5 ♀, 2 ♀ juvenile; Surat Thani Province, Ko Pha-ngan District, Koh Tao Is.; 10°04.07'N, 99°49.16'E; MUMNH-ARA-THE005 • 5 ♀; Loei Province, Phu Ruea District, Lat Khang; 17°31.55'N, 101°15.33'E; MUMNH-ARA-THE007 • 5 ♀; Samut Songkhram Province, Mueang District, Bang Kaeo; 13°23.18'N, 100°02.18'E; MUMNH-ARA-THE008 • 2 ♀; Trat Province, Laem Ngop District, 12°10.38'N, 102°24.33'E; MUMNH-ARA-THE009.

##### Diagnosis.

Sternum black. Sternal band various in shape. Abdomen octagonal, slightly wider than long. Color pattern on dorsal abdomen various but frequently with two large white spots. Three pairs of abdominal spines similar in shape, tubercle with small protuberance at the tip. Ventral side of abdomen black, with scattered yellowish stripes. Ten anterior edge sigilla subequal in size. Four median sigilla arranged in a trapezoid. Ten posterior edge sigilla, the middle pair very small, and close together. Outer posterior edge with five sigilla, located near posterior spines. Epigynum relatively simple in shape with bracket-shaped scape (Fig. 12K). Spermathecae oval, placed close together (Fig. 12J, K). Fertilization duct short, emerging posteriorly from spermathecae (Fig. 12J).

##### Variation.

**Figure 10.**
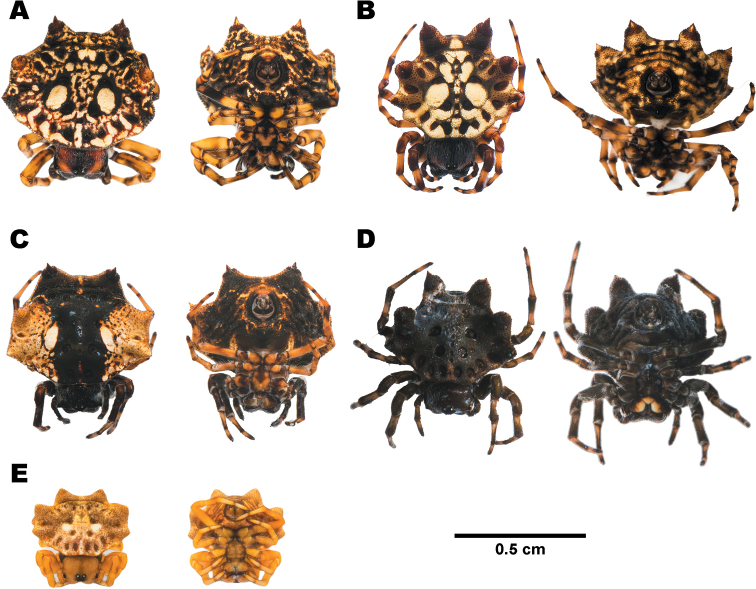
Females of *Thelacantha brevispina* showing dorsal view (left) and ventral view (right) **A, B** multi-color morph **A** specimen from Phetchaburi (MUMNH-ARA-THE004) **B** specimen from Samut Songkhram (MUMNH-ARA-THE008) **C** black-white morph, specimen from Loei (MUMNH-ARA-THE007) **D** black morph, specimen from Samut Songkhram (MUMNH-ARA-THE008) **E** orange morph, specimen from Surat Thani (MUMNH-ARA-THE005).

*Thelacantha brevispina* shows high color variation on abdomen. Four color morphs were found in this study: (1) the multi-colored morph (Fig. 10A, B) is decorated with white, black, and red patches on the dorsal abdomen; (2) the black-white morph (Fig. 10C) possesses a vertical central black line from the anterior to the base of the posterior spines with white areas on each side; (3) the black morph (Fig. 10D) shows a completely black abdomen without the two large white spots; (4) the orange morph (Fig. 10E) is characterized by a bright orange abdomen with two white spots. Such morphotypes are found in adult spiders, except in the orange morph, which was a juvenile specimen. The multi-colored morph was found in every population, whereas the other morphs were relatively rare.

##### Remarks.

*Thelacantha brevispina* has been noted for the two large, distinct white spots on its abdomen (Pickard-Cambridge 1879; Chrysanthus 1959; Emerit 1974; Tikader 1982; Barrion and Litsinger 1995; Yin et al. 1997; Dierkens and Charlat 2011). Some color morphs in this study have been reported in previous works such as the Multi-color morph (Dierkens and Charlet 2011) and the Black-White morph (Tikader 1982). *Thelacantha brevispina* is widely distributed on a global scale. It has been recorded from Madagascar to Australia and also oceanic islands such as French Polynesia, and Fiji (Emerit 1974; Barrion and Litsinger 1995; Dierkens and Charlat 2011). Currently, it is classified as a monotypic species (World Spider Catalog 2020). However, the results of species delimitation have demonstrated four distinct species in the *T. brevispina* lineage (Fig. 4, T1–T4). Worldwide taxon sampling may reveal a large number of cryptic species, and elucidate their taxonomic status.

**Figure 11.**
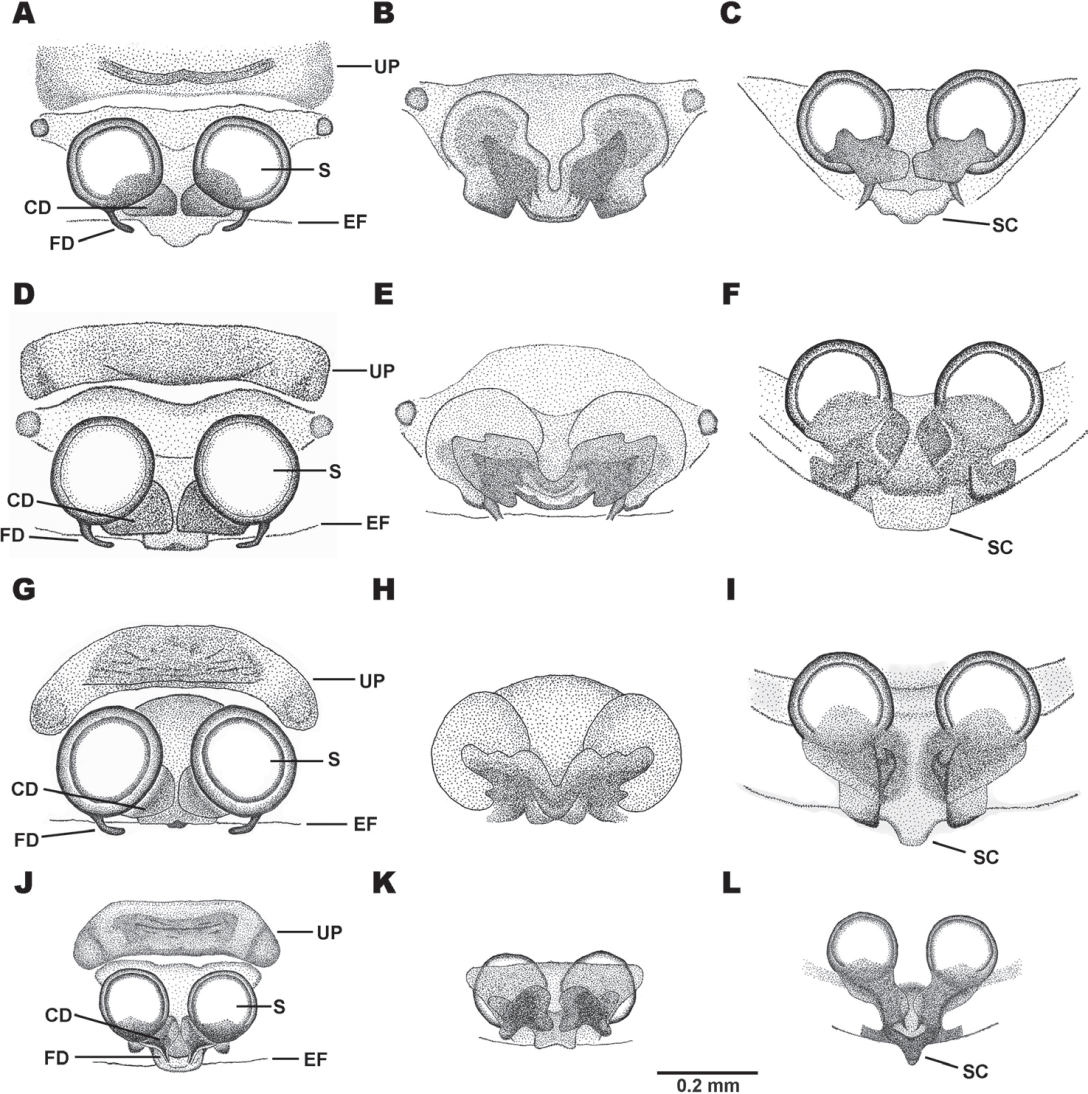
Female genitalia of **A–C***Gasteracantha diadesmia***D–F***G. diardi***G–I***G. doriae***J–L***G. kuhli*. Genitalia are shown in dorsal view (**A, D, G, J**), ventral view from external (**B, E, H, K**), and posterior view (**C, F, I, L**).

##### Distribution and habitat.

India, Pakistan, Bangladesh, Sri Lanka China, Taiwan, Japan, Korea, Myanmar, Thailand, Malaysia, Indonesia (Ambon, Java, Sumatra, and Sulawesi), Philippines, New Guinea, Australia, Fiji, Mauritius, French Polynesia, Hawaii, and Madagascar (Emerit 1974; Tikader 1982; Yin et al. 1997, 2012; World Spider Catalog 2020). In this study, *Thelacantha brevispina* was found widely dispersed in coastal areas. They were commonly found in mangrove forests along the Inner Gulf of Thailand, but one population was found in the mountainous area in Phu Ruea District, Loei Province, which is far from the sea. These spiders build a vertical web between trees in open areas and sit at the center of the web.

**Figure 12.**
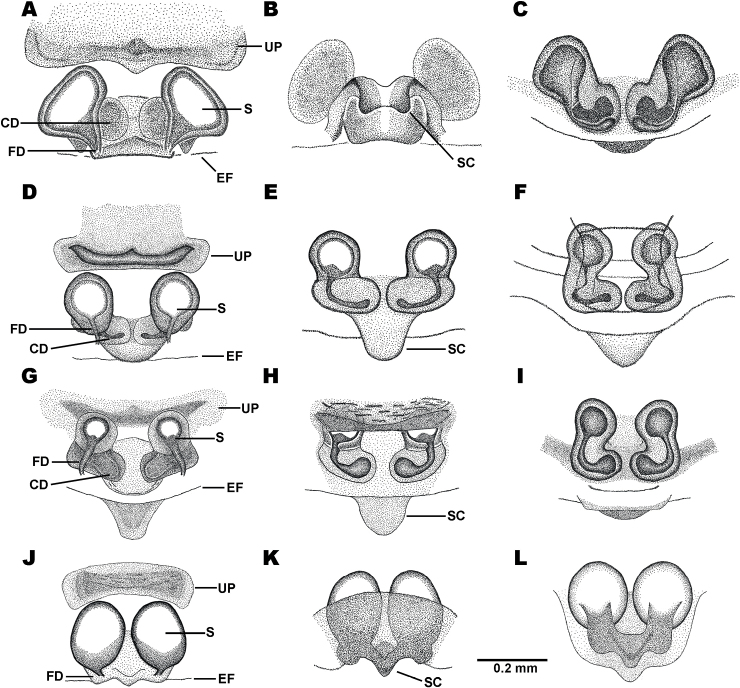
Female genitalia of **A–C***Macracantha arcuata***D–I***M. hasselti***J–L***Thelacantha brevispina*. Genitalia are shown in dorsal view (**A, D, G, J**), ventral view from external (**B, E, H, K**), and posterior view (**C, F, I, L**).

## Discussion

Spiny-backed orb-weaving spiders exhibit high intraspecific variation and also morphological similarities among closely related species (Pickard-Cambridge 1879; Dahl 1914; Chrysanthus 1959; Benoit 1964). Thus, species delimitation is always challenging. This study used molecular approaches to guide the delimitation of species boundaries, and to confirm the morphological classification. The genetic distances based on the COI gene among 15 OTUs show that intraspecific divergence between members of Gasteracanthinae is less than the interspecific divergence, with no overlap between intra- and interspecific distances (Table 3). The gap between intra- and interspecific distance was 1.31–3.76%. The interspecific genetic difference between Gasteracanthinae was 20.55 times than that of the intraspecific genetic difference. This value is higher than the ten times difference originally proposed by Hebert et al. (2004). Moreover, all of the estimates of interspecific genetic distance between species of Gasteracanthinae in this study were greater than 3%, which is the suggested barcoding threshold value for species delineation in arachnids (Barrett and Hebert 2005).

The delimitation results based on the COI gene in all analyses (ABGD, bPTP, and GMYC) confirm 15 distant lineages for the dataset of *Actinacantha*, *Gasteracantha*, *Thelacantha*, and *Macracantha* in the present study. These species delimitation methods are congruent with morphological identification of at least seven examined Thai lineages, consisting of *G. diadesmia*, *G. diardi*, *G. doriae* (D2), *G. kuhli*, *M. arcuata* (M1), *M. hasselti*, and *T. brevispina* (T1). This suggests that the characters of shape and position of abdominal spines, as well as the epigynal structure are useful in delimiting species boundaries in Gasteracanthinae.

In addition, among the 15 discovered lineages, six lineages nested within *T. brevispina*, *M. arcuata*, *G. cancriformis*, and *G. doriae* are likely to be cryptic species (Fig. 4). Apart from the case of *G. doriae*, which has been discussed in the previous taxonomic section, cryptic speciation in other taxa is discussed here. *Thelacantha brevispina* is separated into four different lineages. These lineages are from Thailand (Fig. 4, T1), French Polynesia and Japan (Fig. 4, T3), and two lineages are from Malaysia (Fig. 4, T2 and T4). The clade from Thailand exhibits various color patterns on the abdomen, although their genetic distance is relatively low (0.17%). Furthermore, each color morph is restricted to a single locality, suggesting that each population might have independently evolved their color pattern recently. Also, two specimens of *T. brevispina* from French Polynesia and Japan are grouped into the same lineage; these two islands are geographically distant. This suggests that human activity introduced non-native species from one island to the other (Dawson et al. 2017).

Similarly, *Macracantha arcuata* is separated into two lineages, one from Thailand and Cambodia (Fig. 4, M1), and another from Malaysia (Fig. 4, M2). Deep divergence in both *T. brevispina* and *M. arcuata* corresponds to their geographic distribution. They can be divided into Indochinese (M1, T1) and Sundaic lineages (M2, T2, T4). The biogeographic partition between Indochinese and Sundaic lineages has been observed in other animals such as freshwater shrimp (De Bruyn et al. 2005), amphibians (Emerson et al. 2000), reptiles (Brown, et al. 2012), and birds (Dejtaradol et al. 2016; Manawatthana et al. 2017), as well as in plants (Van Steenis 1950). This phenomenon might suggest a strong paleogeographic barrier between the northern and southern regions of the Southeast Asia mainland (Woodruff 2003, 2010) and/or many colonization events in the area. Two zoogeographical lines, the Isthmus of Kra and the Kangar-Pattani line, are considered as the transition zone between Indochinese and Sundaic biogeographic regions (Woodruff 2003). The results from this study tend to support the Kangar-Pattani line as the boundary line for Gasteracanthinae. However, further model testing and biogeographic study with more samples of Gasteracanthinae from the region should be conducted in order to support our hypothesis.

Deep divergence detected in this study also indicates the possibility of cryptic speciation disguising several species within a nominal name. Unfortunately, we were unable to investigate the type series of *G. cancriformis*, *M. arcuata*, and *T. brevispina*, and topotypes of these species were unavailable, particularly their molecular data. Hence, there was not enough evidence to indicate the taxonomic placement of such distinct lineages. Consequently, we are only able to report such high diversification as a deep divergence within each species.

Based on the phylogenetic tree constructed in this study (Fig. 3), the monophyletic origin of Gasteracanthinae (Fig. 3, node 1) and the great phylogenetic distance between Gasteracanthinae and Micratheninae are congruent with previous studies (Scharff and Coddington 1997; Wheeler et al. 2017; Kallal et al. 2018; Tan et al. 2019; Scharff et al. 2020). Three major clades indicated in the tree are also supported by morphological evidence. Conspecific members in Clade I (Fig. 4, node 2) exhibit metallic bluish black spines, with highly-modified median spines that differ from anterior and posterior spines. They also lack large trapezoid-shaped sigilla on the dorsal abdomen (Tan el al. 2019).

The synapomorphic character common to clade II and III (Fig. 3, node 4) is the presence of a ventral tubercle. Clade III (Fig. 3, node 7) possesses oval spermathecae (Fig. 12J) and six tubercle spines (Fig. 10) as unique characters. In clade II (Fig. 3, node 5), the round spermathecae constitutes a synapomorphic character. The shape of median spines of the broad-abdomen *Gasteracantha* (Fig. 3, node 6) is variable among species, whereas the anterior and posterior spines are similar in shape and direction. They possess large trapezoid-shaped sigilla at the anterior edge, middle, and posterior edges, and small sigilla forming a straight line at the middle of anterior and posterior edges. Taxonomically, their species boundary is difficult to delimit because of morphological similarity (Butler 1873; Pickard-Cambridge 1879; Thorell 1887). Moreover, most broad-abdomen *Gasteracantha* are color polymorphic species, and the horizontal bands morph tends to be conserved within this group. These factors might create confusion for identification. However, the spine character and female genitalia seem to be sufficient to separate the three species of this genus examined in this study. Because this study consists of few members of Gasteracanthinae, further investigation that includes more taxon sampling is needed to indicate phylogenetic relationships among the whole subfamily Gasteracanthinae.

‘*Gasteracantha hasseltii* C. L. Koch, 1837’ has long been placed in genus *Gasteracantha* (World Spider Catalog 2020). However, molecular phylogenetic analysis in this study suggests reclassifying it to genus *Macracantha*. The close phylogenetic relationship between *M. hasselti* and *M. arcuata* is supported by their synapomorphic characters. They share the characteristics of well-developed and elongated median spines, similar pattern of sternal bands, and a concave anterior edge of abdomen. Their posterior edge sigilla are similar in shape and arrangement. Their spinnerets are situated on an elevated black sclerotized structure, forming a shape like a shield volcano (Fig. 9). In the female reproductive organ, the spermathecae of *M. hasselti* and *M. arcuata* exhibit a complex shape (Fig. 12A, D, G), whereas the spermathecae of other Thai *Gasteracantha* in this study are simply round (Fig. 11A, D, G, J). Both species also lack a ventral tubercle, a protuberance between epigynum and spinnerets, while this character is present in other *Gasteracantha* species from Thailand compared in this study. Based on both morphological and molecular-based evidence, it is appropriate to classify these two species in the same genus.

The monophyletic relationship between “*A.*” *globulata* and *Macracantha* is highlighted by the phylogenetic tree in this study with high nodal support. Therefore, it may be appropriate to transfer “*A.*” *globulata* to the genus *Macracantha*. While “*A.*” *globulata* has a distinct characteristic of the tuberculous base of median spines, it also shares morphological characteristics with other *Macracantha* species, i.e., elongated median spines, curved anterior abdomen, sternal band, posterior sigilla that are arranged in a straight line, and the absence of a ventral tubercle (Walckenaer 1841; Hasselt 1882; Tan et al. 2019). Unfortunately, some morphological features of “*A.*” *globulata*, especially the female genitalia structure are still unavailable; only external features of one sub-adult female are illustrated in Tan et al. (2019). Fresh materials of adult females are essential to confirm this hypothesis.

In addition, there are other *Gasteracantha* species that share some morphological characteristics with members of *Macracantha* and potentially should be transferred to the genus, including *Gasteracantha clavatrix* (Walckenaer, 1841), *Gasteracantha clavigera* Giebel, 1863, *Gasteracantha dalyi* Pocock, 1900, *Gasteracantha janopol* Barrion & Litsinger, 1995, *Gasteracantha remifera* Butler, 1873, *Gasteracantha sororna* Butler, 1873. These species exhibit elongated median spines, elevated spinnerets, concave anterior abdomen, and absence of ventral tubercle (Walckenaer 1841; Giebel 1863; Butler 1873; Simon 1877; Pocock 1900; Tikader 1982; Barrion and Litsinger 1995). Their taxonomic placement should be investigated in further study when fresh material of complete adult specimens and their molecular data are available.

Moreover, in this study, the comparative study of abdominal spines in Gasteracanthinae indicated shape variability, especially for a pair of median spines that differ from anterior and posterior spines in many species. The high modification of median spines may have convergently occurred at least twice in clade I and in the clade of broad-abdomen *Gasteracantha*, as well as the for the tubercle spines in *A. globulata* and *T. brevispina*. These examples might be similar to the convergent evolution of long spines in spiny orb-weaving spiders of subfamily Micratheninae, in which the long spine has evolved independently several times within Micratheninae (Magalhaes and Santos 2012). Despite the distant relationship between Micratheninae and Gasteracanthinae, *M. arcuata* (Fabricius, 1793) shows morphological similarity with *Micrathena cyanospina* (Lucas, 1835). Both species possess remarkably long spines, which are very similar in shape (Levi 1985).

## Conclusions

Although intraspecific morphological variation in Gasteracanthinae has been highlighted by some authors (Pickard-Cambridge 1879; Dahl 1914; Kolosváry 1931; Chrysanthus 1959; Benoit 1964; Emerit 1974), our morphological study has demonstrated that the shape and position of abdominal spines, sigilla pattern, and the female genitalia structure are significant characters for species identification and classification. In this study, seven species from three genera, *Gasteracantha*, *Macracantha*, and *Thelacantha*, were identified by both morphological examination and confirmed by molecular approaches. By including previous historical records, we find that there are eleven species of Gasteracanthinae present in Thailand. We transfer ‘*Gasteracantha hasselti*’ to the genus *Macracantha* according to molecular phylogeny and morphological evidence. Most species within Gasteracanthinae exhibit highly intraspecific color polymorphism. Hence, molecular-based analyses provide an applicable tool for indicating species boundaries, and insight into evolutionary history through phylogenetic relationships among taxa. The molecular species delimitation suggests the existence of nine putative species, along with six hidden lineages that seem to be represented as distinct species. Consequently, the number of species in Gasteracanthinae might be underestimated. A comprehensive revision by including more species sampling of both female and male spiders in the future would lead to the discovery more cryptic diversity and lead to a better understanding of the evolutionary history of abdominal spines, intraspecific color polymorphism, sexual dimorphism, as well as phylogeography. These insights will extend the perspectives of colonization patterns of arachnids in Southeast Asia.

## Supplementary Material

XML Treatment for Gasteracantha

XML Treatment for Gasteracantha diadesmia

XML Treatment for Gasteracantha diardi

XML Treatment for Gasteracantha doriae

XML Treatment for Gasteracantha kuhli

XML Treatment for Gasteracantha clavigera

XML Treatment for Gasteracantha frontata

XML Treatment for Gasteracantha irradiata

XML Treatment for Gasteracantha rubrospinis

XML Treatment for Macracantha

XML Treatment for Macracantha arcuata

XML Treatment for Macracantha hasselti

XML Treatment for Thelacantha

XML Treatment for Thelacantha brevispina

## Supplementary materials

Supplementary material 1 Figures S1–S6 Data type phylogenetic trees Explanation note **Fig. S1.** Maximum parsimonious phylogenetic tree reconstructed from COI+16S+H3 dataset. **Fig. S2.** Bayesian inference phylogenetic tree reconstructed from COI+16S+H3 dataset. **Fig. S3.** Maximum parsimonious phylogenetic tree reconstructed from COI gene. **Fig. S4.** Maximum likelihood phylogenetic tree reconstructed from COI gene. **Fig. S5.** Ultrametric tree reconstructed from 454 bp of 16S gene showing clusters of OTUs as suggested by morphological identification, and three molecular species delimitation algorithms. **Fig. S6.** Ultrametric tree reconstructed from 328 bp of H3 gene showing clusters of OTUs as suggested by morphological identification, and three molecular species delimitation algorithms. https://binary.pensoft.net/file/532446This dataset is made available under the Open Database License (http://opendatacommons.org/licenses/odbl/1.0/). The Open Database License (ODbL) is a license agreement intended to allow users to freely share, modify, and use this Dataset while maintaining this same freedom for others, provided that the original source and author(s) are credited.Kongkit Macharoenboon, Warut Siriwut, Ekgachai Jeratthitikul
