# Supplementary material for: A review of the taxonomy of spiny-backed orb-weaving spiders of the subfamily Gasteracanthinae (Araneae, Araneidae) in Thailand
Source: Zookeys. 2021 Apr 16;1032:17–62. doi: 10.3897/zookeys.1032.62001 (PMC8065025; doi:10.3897/zookeys.1032.62001)
Supplement: Supplementary material 1 — Figures S1–S6 [file zookeys-1032-017-s001.pdf]

Supporting Materials

A review of the taxonomy of spiny-backed orb-weaving spiders of the subfamily Gasteracanthinae (Araneae: Araneidae) in Thailand

Kongkit Macharoenboon, Warut Siriwut, and Ekgachai Jeratthitikul

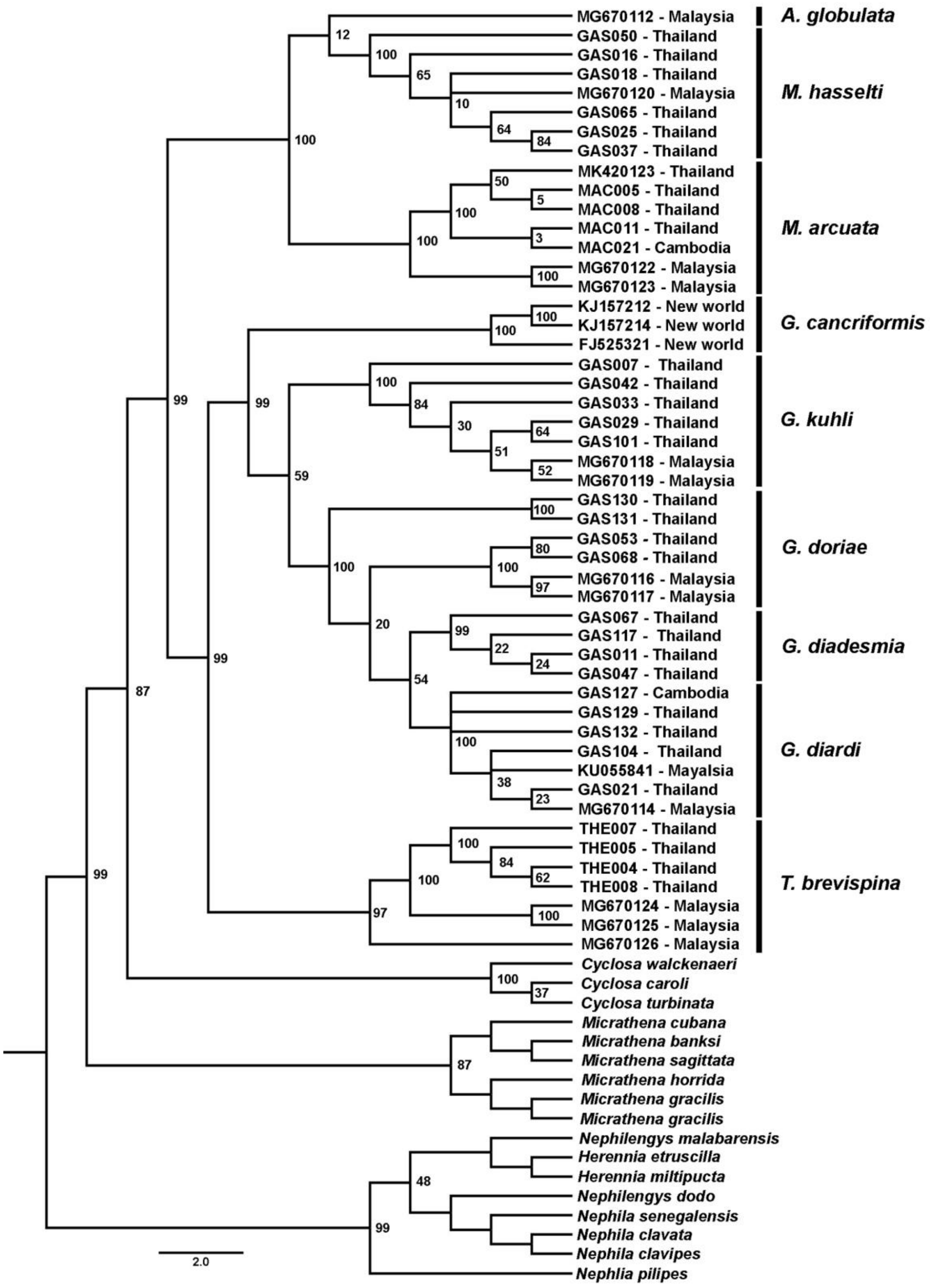

Figure S1 Maximum Parsimonious phylogenetic tree reconstructed from COI+16S+H3 genes of Gasteracanthinae and outgroups. Nodal support values are labeled as Jackknife support.

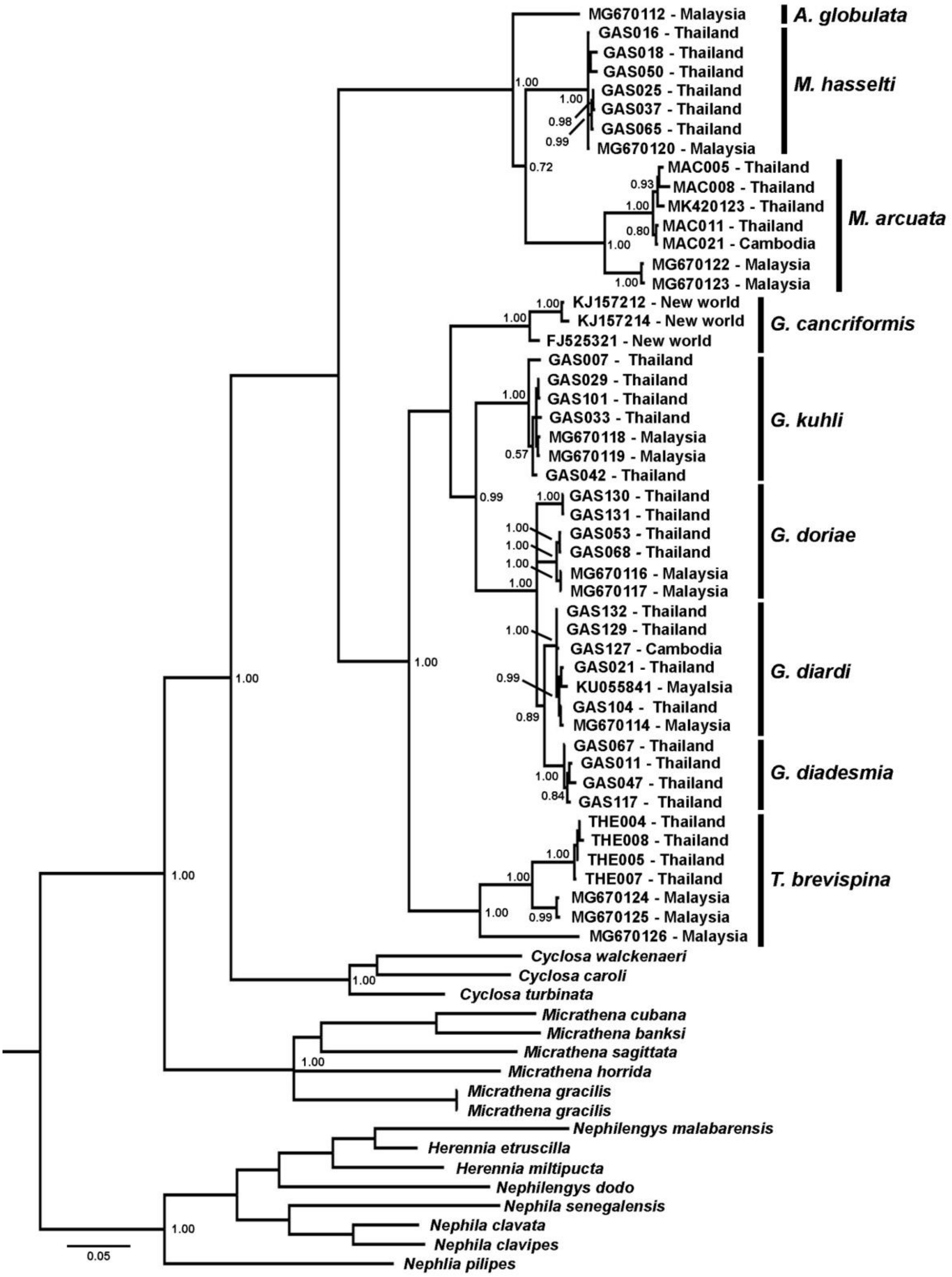

Figure S2 Bayesian inference phylogenetic tree reconstructed from COI+16S+H3 genes of Gasteracanthinae and outgroups. Nodal support values are labeled as Bayesian posterior probability.

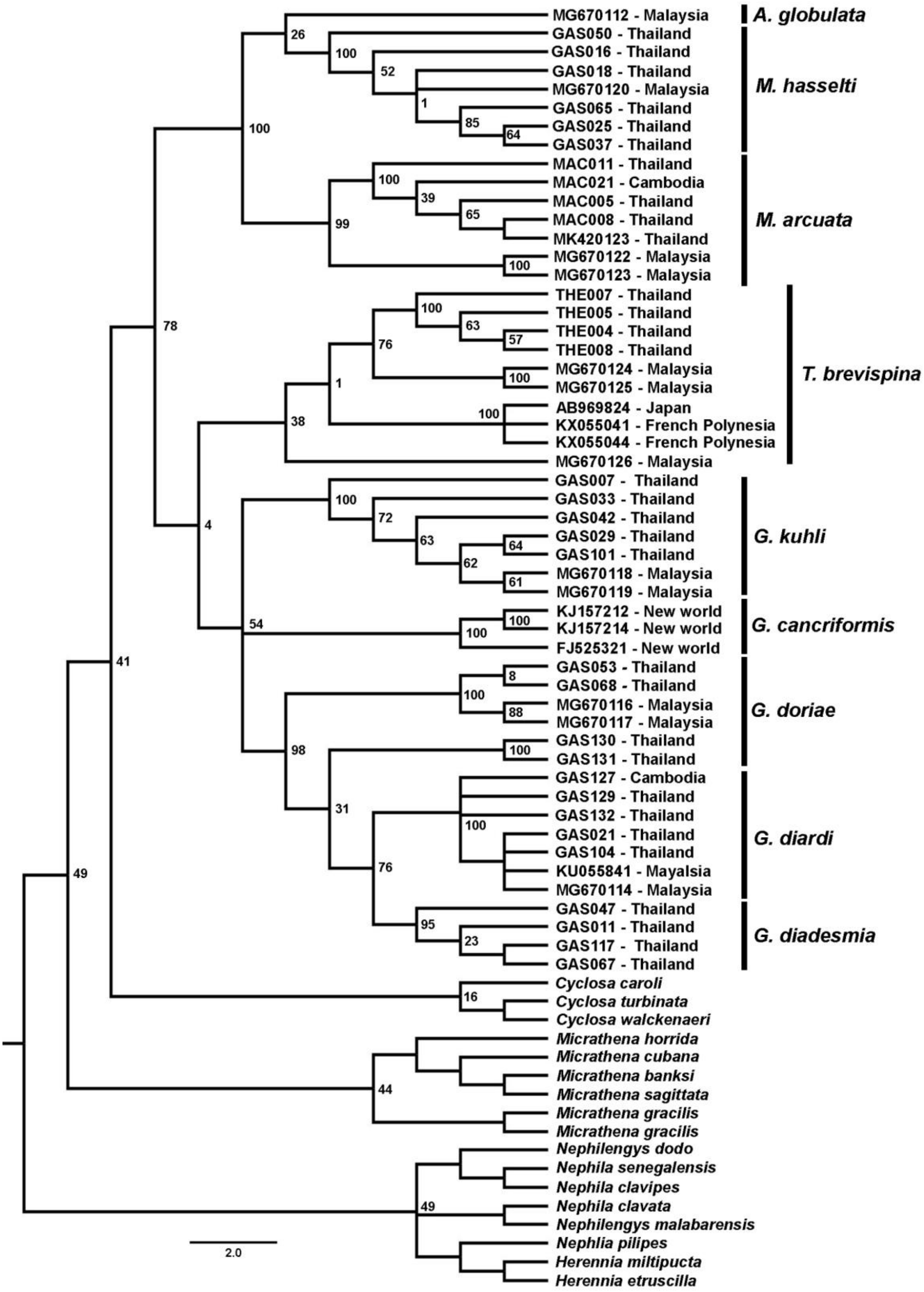

Figure S3 Maximum parsimonious phylogenetic tree reconstructed from COI gene of Gasteracanthinae and outgroups. Nodal support values are labeled as Jackknife support.

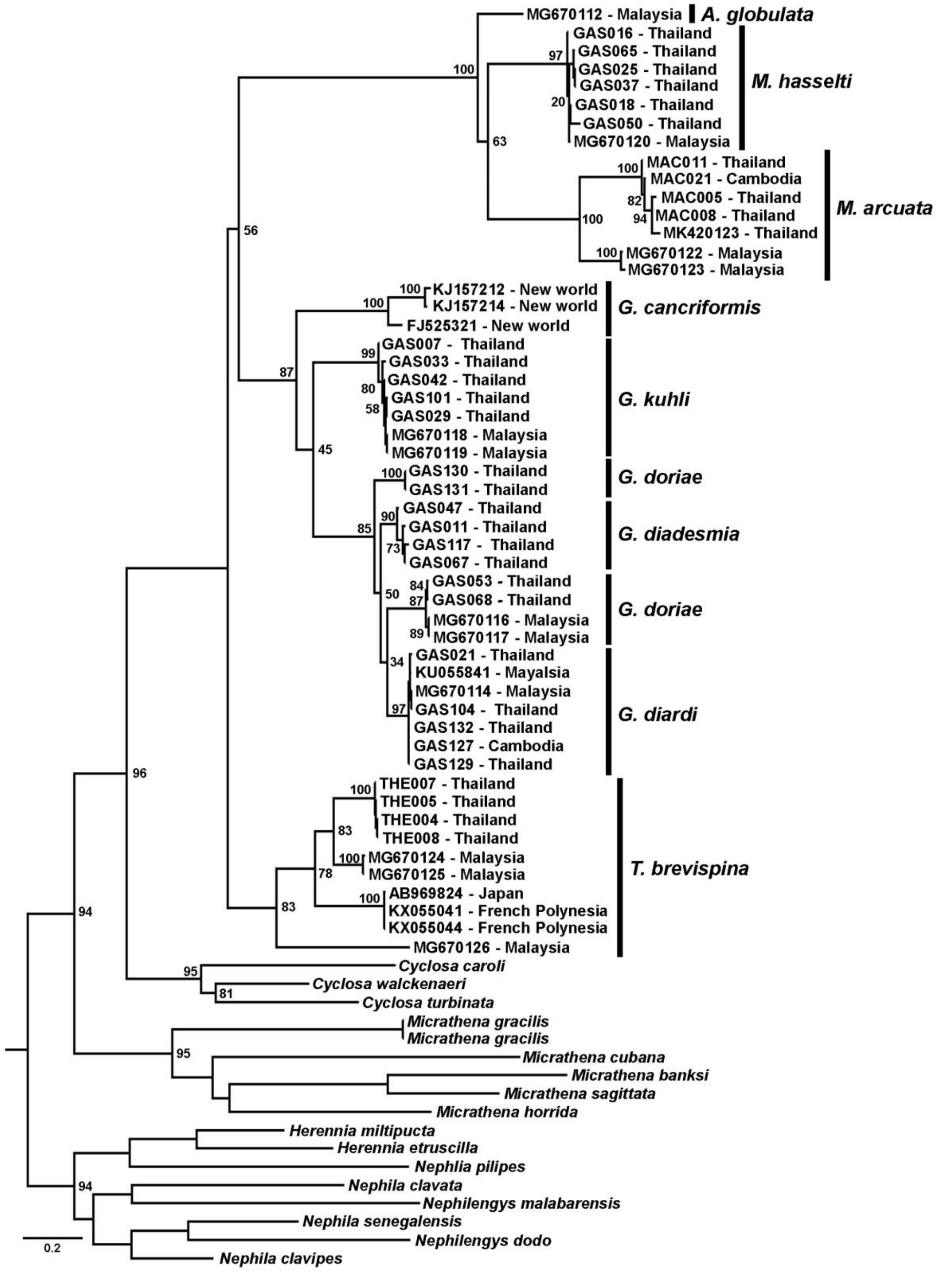

Figure S4 Maximum likelihood phylogenetic tree reconstructed from COI gene of Gasteracanthinae and outgroups. Nodal support values are labeled as ML bootstrap values.

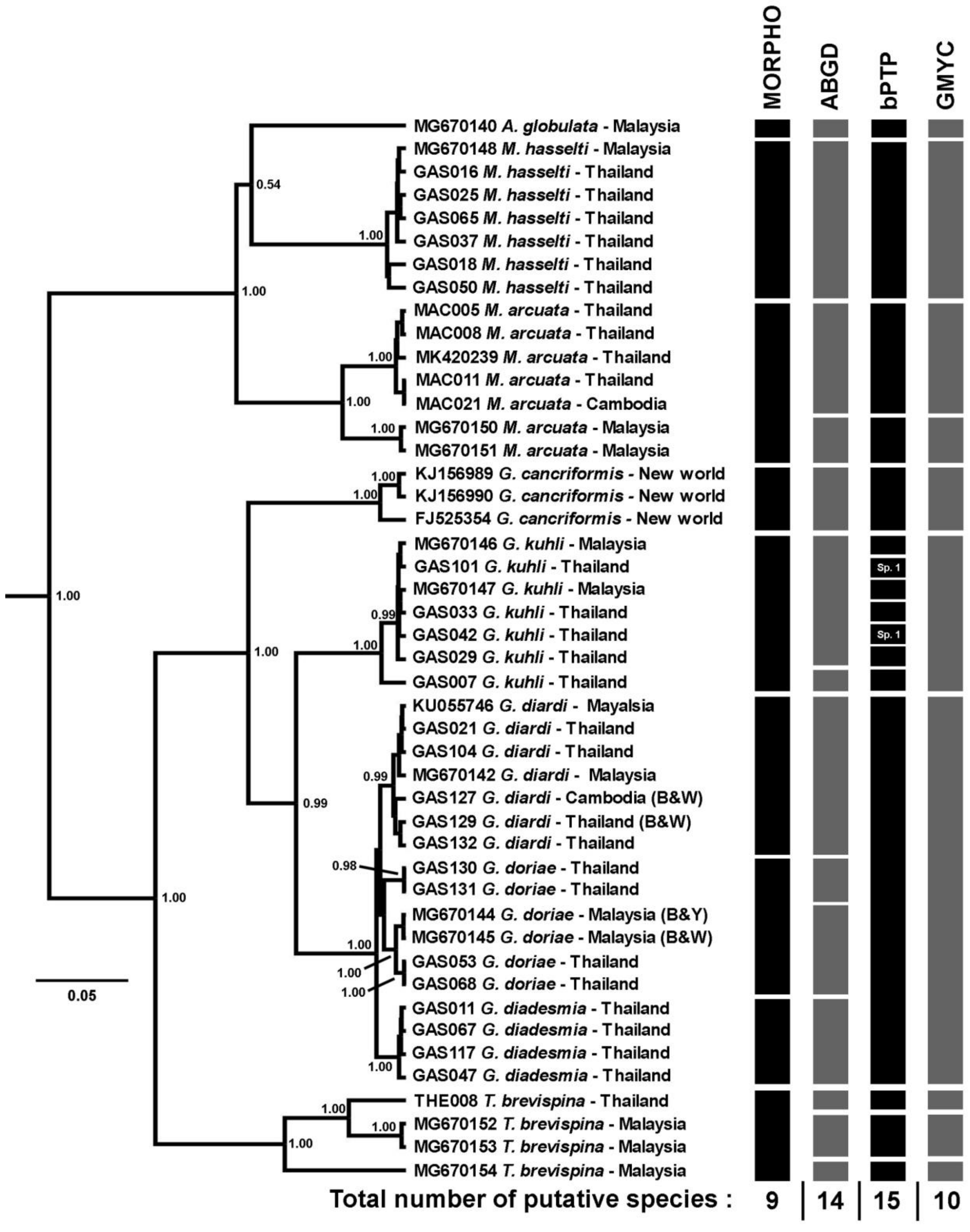

Figure S5 Ultrametric tree reconstructed from 454 bp of 16S gene showing clusters of OTUs as suggested by morphological identification, and three molecular species delimitation algorithms. Nodal support values are labeled as Bayesian posterior probability.

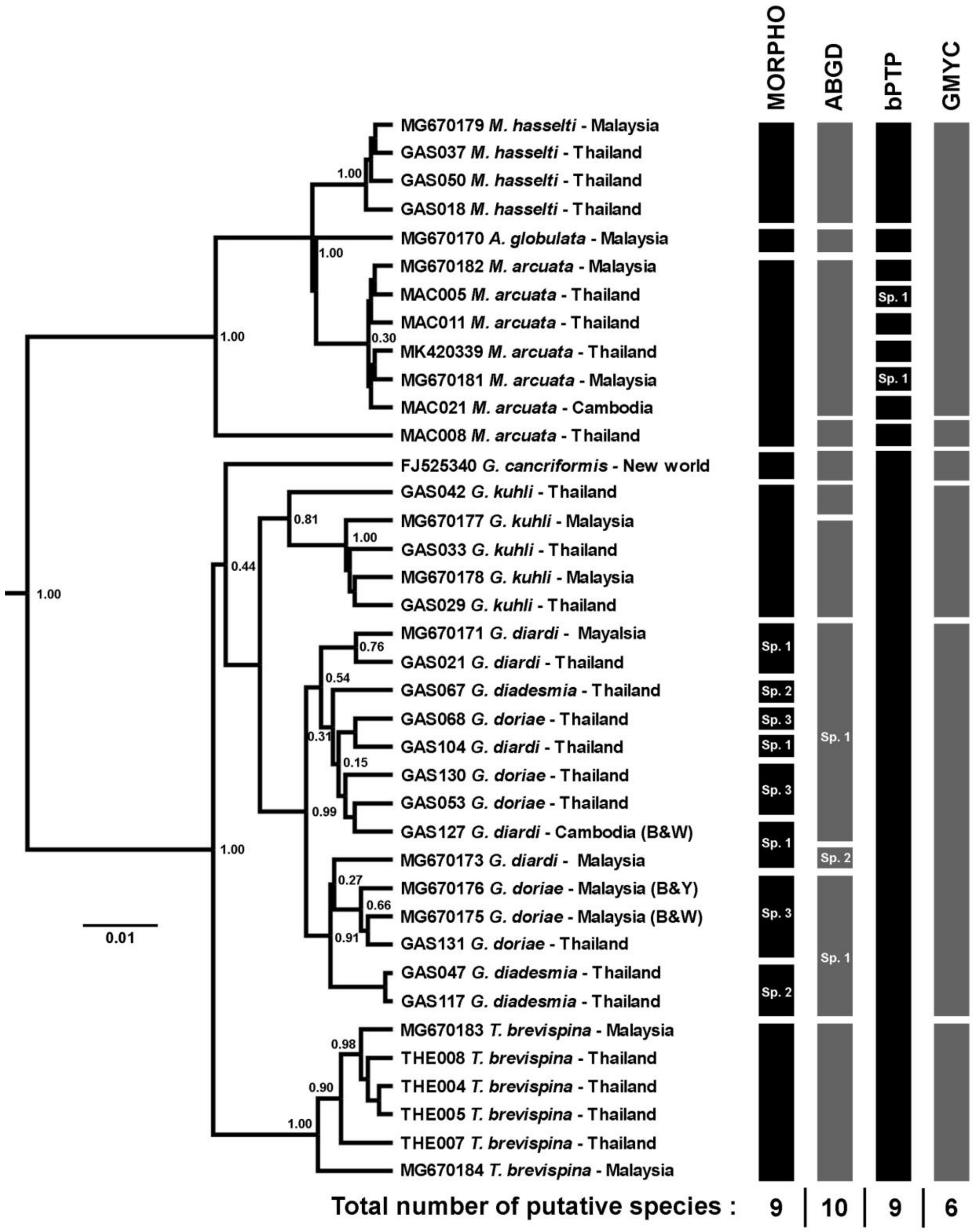

Figure S6 Ultrametric tree reconstructed from 328 bp of H3 gene showing clusters of OTUs as suggested by morphological identification, and three molecular species delimitation algorithms. Nodal support values are labeled as Bayesian posterior probability.
